# Protective factors for maternal mental health and life satisfaction during the COVID-19 pandemic: a longitudinal analysis

**DOI:** 10.1136/bmjopen-2025-110204

**Published:** 2026-01-27

**Authors:** Pia Myklebust Johannessen, Christian Madsen, Rannveig Kaldager Hart, Ingunn Olea Lund, Johanne Hagen Pettersen, Kristin Gustavson, Espen Røysamb, Ragnar Nesvåg, Ragnhild Eek Brandlistuen, Helga Ask

**Affiliations:** 1 PsychGen Center for Genetic Epidemiology and Mental Health, Norwegian Institute of Public Health, Oslo, Norway; 2 Department of Psychology, University of Oslo, Oslo, Norway; 3 Department of Disease Burden, Norwegian Institute of Public Health, Bergen, Norway; 4 Centre for Disease Burden, Norwegian Institute of Public Health, Bergen, Norway; 5 Department of Health Management and Health Economics, University of Oslo, Oslo, Norway; 6 Centre for Fertility and Health, Norwegian Institute of Public Health, Oslo, Norway; 7 Department of Child Health and Development, Norwegian Institute of Public Health, Oslo, Norway; 8 PROMENTA Research Center, University of Oslo, Oslo, Norway; 9 Division of Public Health and Prevention, Norwegian Institute of Public Health, Oslo, Norway

**Keywords:** COVID-19, MENTAL HEALTH, PUBLIC HEALTH, Longitudinal studies

## Abstract

**Abstract:**

**Objective:**

Mothers’ mental health and life satisfaction may have been negatively affected due to challenges during the COVID-19 pandemic. Given the risk of future crises, knowledge of possible mitigating factors in this population is essential. This study aims to examine whether the pandemic affected the level of protective factors such as social support, physical activity and employment situation, and how these factors are associated with mental distress and life satisfaction.

**Design:**

Longitudinal cohort study.

**Outcome measures:**

Primary outcomes were mental distress (measured by the eight-item version of the Hopkins Symptom Checklist) and life satisfaction (measured by the Satisfaction With Life Scale). As the first step, we investigated changes in the levels of social support (defined by the number and frequency of social contact), physical activity (average hours of physical activity during a week), employment situation (actively working vs sick leave or unemployed), alcohol consumption (measured by the Alcohol Use Disorders Identification Test-Consumption) and relationship satisfaction (measured by the five-item version of the Relationship Satisfaction Scale).

**Methods:**

We analysed data from two waves of the Norwegian Mother, Father and Child Cohort Study (n=~18 000 mothers); one pre-pandemic wave and one wave where half of the sample responded after the onset of the pandemic, with pandemic exposure being defined by questionnaire response timing rather than cohort recruitment. To assess changes in protective factors over time and pandemic exposure, we used difference-in-differences analyses and regression discontinuity design. Associations between protective factors with mental distress and life satisfaction, and possible moderation by pandemic exposure, were investigated using multiple regression models with interaction terms adjusted for potential confounders.

**Results:**

Apart from physical activity, which declined less across time in the pandemic group (B=0.09, 99% CI 0.05 to 0.12), protective factors did not change during the pandemic. Social support, employment situation and relationship satisfaction were associated with mental distress and life satisfaction, whereas physical activity showed a unique relationship with mental distress. Most associations were similar across pandemic exposure groups, except employment situation which appeared to have a stronger protective effect in the pandemic group (β=−0.12, 99% CI −0.24 to −0.00).

**Conclusions:**

Changes over time in self-reported levels of protective factors were generally consistent among mothers independent of the pandemic. These factors appear to play an equally important role for mental distress and life satisfaction both under ordinary circumstances and during public health crises. Our findings enhance the understanding of how potential protective factors among mothers are associated with mental distress and life satisfaction in the context of a global stressor. Future studies should investigate additional mitigating factors that may be particularly relevant during global crises and explore the causal relationship between protective factors, mental health and life satisfaction.

**STRENGTHS AND LIMITATIONS OF THIS STUDY**

- This study used data from more than 18 000 mothers participating in a large population-based prospective birth cohort study in Norway.
- Although robust statistical methods were employed, the study design does not allow for conclusions about causal direction.
- Potential biases may arise from the use of self-reported measures and the relatively low response rate, both at initial recruitment and in the follow-up survey conducted when the child was approximately 14 years old, which may limit the generalisability of the findings.

## Introduction

 The COVID-19 pandemic created profound disruptions for parents, particularly mothers. For instance, school closures intensified family strain, while mothers also reported low co-parent involvement, increased gender imbalance in household duties and increased work-home conflict.^1^ Such factors might affect mental health, often conceptualised as comprising two related but distinct dimensions; well-being and ill-being.^2^ Well-being typically includes aspects of subjective well-being,^3^ such as life satisfaction, defined as the cognitive evaluation of one’s life as a whole,^4^ while ill-being includes symptoms of mental illness,^5^ ranging from mental distress to mental disorders. During the pandemic, studies show that mothers reported heightened mental distress^6^ and more substantial declines in life satisfaction compared with fathers.^7^ This disparity underscores the need to identify protective factors that might mitigate mental health challenges and enhance life satisfaction among mothers.

The emergence of resilience research and later positive psychology in the mid-1990s marked a shift in the scientific literature from symptom-focused medical models to positive outcomes and protective factors.^8^ Drawing on Bronfenbrenner’s ecological systems theory,^9^ protective factors can be understood as resources within various levels of an individual’s ecological environment that reduce negative effects or enhance positive outcomes. Existing literature highlights several protective factors for mental health, including social support, physical activity, employment, lower alcohol use and relationship satisfaction.^10^,^12^ These protective factors are also anchored in other established theoretical frameworks. For example, the Transactional Theory of Stress and Coping^13 14^ highlights the role of emotion- and problem-focused coping, which could include using social support, physical activity and reducing alcohol intake to regulate emotions and handle stress, while the family resilience framework^15 16^ emphasises relational processes such as relationship satisfaction to help families adapt to adversity. However, the pandemic may have disrupted such protective factors. For example, mothers reported reduced physical activity^17 18^ and increased experiences of job loss or anticipated job loss^19^ during the pandemic. Evidence regarding social support,^20 21^ alcohol use^22^ and relationship satisfaction^23 24^ among mothers is mixed, potentially due to variations in child age, timing of data collection across different pandemic phases and differences in the measurement instruments used.

Understanding how protective factors relate to maternal mental health and life satisfaction during a crisis like the COVID-19 pandemic is critical. Previous research, both among mothers and in the general population, has established that social support was associated with fewer symptoms of anxiety and depression,^25 26^ and higher life satisfaction.^27^ Likewise, reduced physical activity levels during the pandemic were associated with worse mental health^28^ and lower life satisfaction.^29^ Employment situation among mothers also plays a central role and is associated with both mental health^19^ and life satisfaction^30^ during the pandemic.

Further, the association between alcohol use during the pandemic and mental health among parents has been heterogenous.^22^ Similar inconsistencies have been observed for the association between relationship quality or couples’ satisfaction and mental health among parents during the pandemic.^28 31^

Importantly, much of the existing research has focused on perinatal or postpartum mothers, leaving a gap in understanding mothers of older children. The developmental stages of childhood and adolescence introduce distinct challenges compared with early parenthood. As children mature, increasing autonomy, academic demands and social complexity can heighten parental stress and require different coping strategies. Mothers at these stages often balance caregiving with employment and household responsibilities, making protective factors such as social support, relationship satisfaction and behavioural regulation particularly relevant. Additionally, few studies have used life satisfaction as a key outcome alongside mental distress when investigating pandemic effects among mothers, and many lack pre-pandemic baseline measures, limiting the ability to assess changes over time.

## Objectives

Our study contributes to the literature by analysing longitudinal data from more than 18 000 mothers in the Norwegian Mother, Father and Child Cohort Study (MoBa), collected before, during and after the pandemic. As culture, society and national policies may influence which factors are important to mental distress and life satisfaction, our study adds to the understanding of potential protective factors in Norway, an egalitarian country with a highly developed social welfare system. We address two primary research questions:

1. Assessing change in protective factors: How did the levels of five putative protective factors (social support, physical activity, employment situation, alcohol consumption and relationship satisfaction) change during the pandemic compared with pre-pandemic times?
2. Evaluating associations between protective factors and mental health and life satisfaction: How are these protective factors associated with mental distress and life satisfaction, and do these associations differ between mothers exposed to the pandemic and those who were not?

## Methods

### Preregistration

This article has been preregistered in the open science framework (https://osf.io/4zvpj/overview?view_only=). See online supplemental table 1 in Appendix for a list of deviations, which mostly are specifications of data and variables used and choices regarding the number of analyses conducted.

### Study design and sample

MoBa is a population-based pregnancy cohort study conducted by the Norwegian Institute of Public Health.^32^ Participants were recruited from all over Norway from 1999 to 2008. The women consented to participation in 41% of the pregnancies. The cohort includes approximately 114 500 children, 95 200 mothers and 75 200 fathers.

The establishment of MoBa and initial data collection was based on a licence from the Norwegian Data Protection Agency and approval from The Regional Committees for Medical and Health Research Ethics. The MoBa cohort is currently regulated by the Norwegian Health Registry Act.

We linked MoBa data to the Medical Birth Registry of Norway (MBRN) and Statistics Norway. MBRN is a national health registry containing information about all births in Norway, and Statistics Norway is the main producer of official statistics in Norway.

Two waves of data collection were used: when the child was 8 years old (Q8, n=43 483 mothers) and 14 years old (Q14, n=26 492 mothers). The response rates were 46.7%^33^ for Q8 and 32.0% for Q14.^34^ Only responses from the first-born MoBa child were included for each mother to avoid dependency between observations.

Two analytical samples were defined based on the statistical analyses used to examine the two research questions. Both samples were restricted to mothers responding to Q8 and Q14 (n=18 339, sample 2). In order to examine within-person pandemic effects on protective factors, sample 1 (n=18 015) was further restricted to make the data waves more comparable. Due to a delay in the initiation of the Q14 data collection, Q14 data were collected over a 7-year period (2017–2023). Consequently, we also restricted Q8 data to responses collected between 2011 and 2017 (7 years). Q14 mothers who (due to the delay) responded when their child was older than 15 years old in Q14 were excluded from sample 1.

### Measures

#### Mental health outcomes

Mental distress was measured by the eight-item version of the Hopkins Symptom Checklist (SCL-8).^35^ Each item is scored on a four-point Likert scale, ranging from 1 (‘not bothered’) to 4 (‘very bothered’), and mean scores were calculated for individuals that filled out at least four of the items. Life satisfaction was assessed by The Satisfaction With Life Scale (SWLS).^4^ The five items are scored on a seven-point Likert scale, ranging from 1 (‘strongly disagree’) to 7 (‘strongly agree’). Mean scores were calculated for individuals that filled out at least two of the five items.

For more details about the preparation of the mental health outcomes, see online supplemental file 1, Appendix section 2.

#### Protective factors

Two items assessed social support, measuring both availability (having someone other than a partner to seek advice from in a difficult situation) and frequency (how often mothers meet or talk with their extended family or close friends). Each item was scored on a three-point scale, ranging from 1 to 3. Mean scores were calculated for individuals responding to at least one of the items. Frequency of physical activity, defined as activities in which you become short of breath or sweat, was assessed by three items we converted into separate subscales indicating the total hours of exercising for up to 30 min, 30–60 min and more than 60 min during a week (see details in online supplemental file 1, Appendix section 2). We combined responses on the subscales to create a variable indicating the mean hours of physical activity during the week. Mean scores were calculated for individuals with information on at least one of the three subscales.

The employment situation was assessed by the item: ‘Are you currently in paid employment (full or part time)?’. The response options were ‘Yes’, ‘Yes, but currently on sick leave’ or ‘No’. Based on the responses, we created two different binary variables to investigate change over time: currently unemployed versus employed and currently on sick leave versus employed. When examining the potential protective effect of being actively working on levels of mental distress and life satisfaction in the regression models, we created a binary variable separating those currently employed versus those on sick leave/unemployed. Alcohol consumption was measured by the Alcohol Use Disorders Identification Test-Consumption (AUDIT-C),^36^ which consists of three items scored from 0 to 4. In this study, AUDIT-C was treated as a continuous variable, based on the mean of the responses. Mean scores were calculated for individuals who had filled in at least one of the three items.

Relationship satisfaction was measured by the five-item version of the Relationship Satisfaction Scale (RS5).^37^ Each item was scored on a six-point scale with response options ranging from ‘disagree completely’ (1) to ‘agree completely’ (6). Mean responses were calculated for participants that filled in at least two of the five items, and additionally for participants with at least one item filled in.

For more details about the preparation of the protective factors, see online supplemental file 1, Appendix section 2.

#### Pandemic groups

We divided mothers into pre-pandemic and pandemic groups, based on the timing of response to Q14. For analyses using life satisfaction as outcome, those responding before the Norwegian Lockdown (12 March 2020) were classified as pre-pandemic and those responding later were classified as the pandemic group. Due to the 2 weeks reference period of SCL-8, the definition of the pre-pandemic and pandemic group was set as before or after 26 March 2020 in analyses using mental distress as outcome.

#### Time

Time was measured by three different variables: (1) a centred time variable counting months before and after the Norwegian lockdown, (2) a binary variable, coded 1 or 2 to indicate wave of data collection (Q8=1, Q14=2) and (3) a variable with the date of the Q14 response.

#### Covariates

Covariates included mothers’ age at Q8 and Q14 (from MBRN), highest level of education in 2020 (from Statistics Norway), and whether mothers shared household with others (eg, partner, other children, etc) in addition to the child (from Q8). In the regression analyses, we also included a baseline measure (from Q8) of the outcome of interest and a time variable indicating the specific date the mothers responded to Q14 as covariates in the model.

### Statistical analyses

Simple difference-in-difference (DID) analyses were used to investigate change in the protective factors (social support, physical activity, employment situation and alcohol consumption) (see online supplemental file 1, Appendix section 5.1 for more information). DID analyses evaluate the effect of an event (ie, the COVID-19 lockdown) by comparing the change in one group (ie, the pandemic group) before (ie, Q8) and after (ie, Q14) the event to the change over the same number of years in the unexposed (ie, pre-pandemic) group.^38^ The regression model was fitted using sample 1. SEs were robust and clustered at the individual level.

Due to the lack of a Q8 measure for relationship satisfaction, we used regression discontinuity design (RDD) to investigate an immediate change in level of relationship satisfaction between the pre-pandemic and pandemic groups at the Norwegian lockdown (see details in online supplemental file 1, Appendix section 5.2). In RDD, the exposure to an event (ie, the COVID-19 lockdown) is based on a cut-off point (ie, 12 March 2020) on a continuously measured running variable (ie, time centred at lockdown).^39^ SEs were estimated using heteroskedasticity-robust SEs.

To estimate the associations of the five putative protective factors with mental health outcomes, we used multiple regression analyses on sample 2. Mental distress and life satisfaction were included as outcomes in separate models, with protective factors and pandemic group as predictors. Interaction terms between protective factors and pandemic exposure were included to estimate whether the associations differed depending on pandemic exposure. All outcomes and predictors were standardised to have a mean of 0 and SD of 1.

All models were run with and without adjustment for potential endogenous confounding factors. The significance level in all analyses was set to 0.01, with corresponding 99% CI. A conservative p value threshold was chosen, to reduce the risk of type I errors due to multiple comparisons.

Due to a large sample size and no major differences between the analytic sample and the full MoBa sample (see online supplemental table S3 in Appendix), we used complete-case analysis. This approach assumes that missingness is not strongly related to the outcomes conditional on observed covariates, and sensitivity analyses yielded similar results. All analyses were conducted in RStudio with R V.4.1.2.^40^ All figures were generated using the ggplot2 package.^41^ Regression models were conducted using the lm() function in base R.

### Sensitivity analyses

Participants who did not meet our criteria for calculating mean scores (ie, information on at least 50% of items in a scale) were excluded from the analyses (see online supplemental table S2 in Appendix for information about missing data). We repeated the regression analyses including only mothers without item-level missing data on the outcome variables. Another set of sensitivity analyses was run, including interaction terms with the Q8-year data as a negative control (where both groups responded before the pandemic). AUDIT-C showed a low reliability, which might indicate that the items included in AUDIT-C measure different aspects of alcohol consumption. Despite low reliability, we still believe that the total score of these different aspects provides a useful measure of alcohol consumption. However, to test whether the low reliability led to an underestimation of the associations, we performed separate regression analyses including only the first item as predictor. We also did various sensitivity tests to check the robustness of the RDD result (see online supplemental file 1, Appendix section 5.1.2 for more information regarding the various RDD robustness tests and R packages used).

### Patient and public involvement

The same representatives from mental health user organisations have been involved with the overarching research project since the grant-application stage, with regular meetings throughout the project period. For the present study, representatives were not involved in study design, selection of outcomes, recruitment, data collection or assessment of participant burden. They were involved during the interpretation of the findings and dissemination planning. Together with user organisations, we co-designed an open seminar where results were presented, alongside contributions from four mental health user organisations. We also presented the results at a meeting with >10 mental-health user organisations, in a format agreed on with user organisations.

## Results

Descriptive statistics for the analytic samples are presented in table 1. For comparison with characteristics in the full MoBa sample, see online supplemental table S3 in Appendix.

**Table 1:** Demographic information describing the two samples

		Sample 1 (n=18 015)	Sample 2 (n=18 339)
Mean age Q8 (SD)		36.1 (4.4)	–
Mean age Q14 (SD)		43.0 (4.5)	43.0 (4.5)
Living situation Q8, n (%)	Alone with the child	1468 (8.1)	1493 (8.1)
	Together with others and the child	16 281 (90.4)	16 573 (90.4)
Education,* n (%)	Compulsory	401 (2.2)	412 (2.2)
	Upper secondary	3310 (18.4)	3362 (18.3)
	Bachelor	9993 (55.5)	10 191 (55.6)
	Master	3891 (21.6)	3946 (21.5)
	PhD	409 (2.3)	416 (2.3)
Pandemic group, n (%)	Pre-pandemic (before12 March 2020)	7590 (42.1)	7798 (42.5)
	During pandemic (on or after12 March 2020)	10 425 (57.9)	10 541 (57.5)

* Information from Statistics Norway regarding the highest attained education in 2020.

### Changes in protective factors before and after the onset of the COVID-19 pandemic

**Figure 1:**
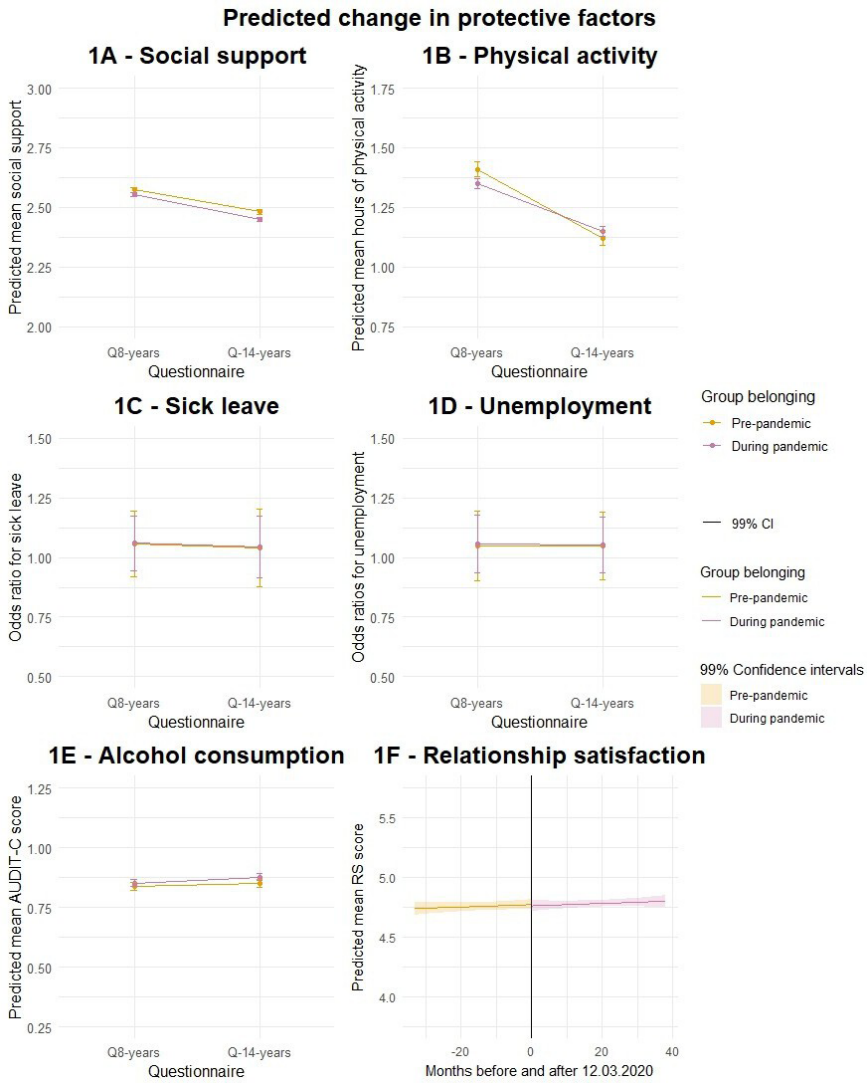
Difference-in-differences models (**A, B, C, D, E**) and RDD model (**F**) showing the pandemic effect on the predicted change in protective factors among mothers in the pre-pandemic (orange colour) and pandemic (pink colour) groups, adjusted for age, education and living situation with 99% CI. Panels A, B, E and F show regression coefficients, and panels C and D show ORs. AUDIT-C, Alcohol Use Disorders Identification Test-Consumption; RDD, regression discontinuity design; RS, Relationship Satisfaction Scale.

The main findings from the adjusted DID (and adjusted RDD for relationship satisfaction) analyses are presented in figure 1, with unadjusted and adjusted parameter estimates presented in online supplemental tables S4–S9 in Appendix.

There was no pandemic effect on the level of social support, as the change from Q8 to Q14 was similar for the pandemic and pre-pandemic group (B=−0.013, 99% CI −0.032 to 0.005, see figure 1, panel A). Among all mothers, there was a general decrease in social support from Q8 to Q14 (B=−0.092, 99% CI –0.106 to –0.078). The pandemic group reported a lower level of social support at Q8 compared with the pre-pandemic group (B=−0.019, 99% CI –0.037 to –0.001).

We found a pandemic effect on the amount of physical activity over time when comparing the pre-pandemic and pandemic group (B=0.085, 99% CI 0.046 to 0.124 see figure 1, panel B), as the change from Q8 to Q14 was different between the two groups. The pandemic group went from exercising less than the pre-pandemic group at Q8 (B=−0.057, 99% CI –0.096 to –0.019) to exercising more than the pre-pandemic group at Q14. In addition, we found a general time effect, as physical activity decreased from Q8 to Q14 among all mothers (B=−0.281, 99% CI –0.311 to –0.251).

There was no pandemic effect in the level of sick leave (OR=1.085, 99% CI 0.837 to 1.408) and no group difference at Q8 (OR=1.060, 99% CI 0.891 to 1.262, see figure 1, panel C). However, we found a small significant time effect, as the odds of being on sick leave were 31.8% lower in Q14 than in Q8 (OR=0.682, 99% CI 0.558 to 0.833). Regarding unemployment (see figure 1, panel D), we found neither a pandemic effect (OR=0.950, 99% CI 0.774 to 1.167), group difference at Q8 (OR=1.141, 99% CI 0.955 to 1.364), nor time effect (OR=1.000, 99% CI 0.855 to 1.169).

We found neither a pandemic effect on alcohol consumption (B=0.011, 99% CI –0.004 to 0.028, see figure 1, panel E) nor a group difference at Q8 (B=0.015, 99% CI –0.005 to 0.035). However, we found a small significant time effect, as alcohol consumption increased from Q8 to Q14 among all mothers (B=0.013, 99% CI 0.001 to 0.025).

There was no immediate pandemic effect on the level of relationship satisfaction at lockdown when comparing the pre-pandemic and pandemic groups (B=−0.010, 99% CI –0.086 to 0.065, see figure 1, panel F). There was neither a time effect before nor after lockdown (B=0.001, 99% CI –0.001 to 0.003). The robustness tests further confirmed the absence of a pandemic effect at lockdown (see online supplemental tables S20 and S11 in Appendix).

### Association between protective factors and mental health outcomes

**Figure 2:**
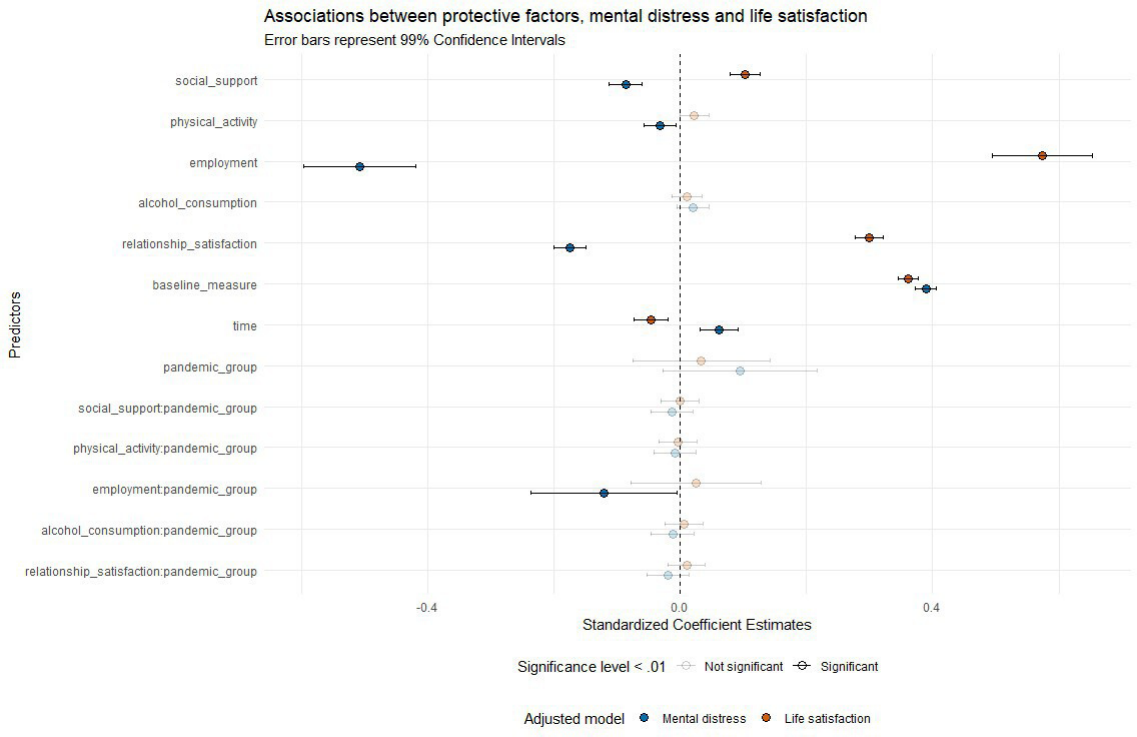
Associations between protective factors and mental distress (blue colour) or life satisfaction (red colour), adjusted for age and education, in addition to baseline symptom levels and time, with 99% CI. The x-axis shows standardised coefficients. Exact standardised coefficients and CIs are provided in online supplemental tables S12 and S13 in the Appendix.

Associations between protective factors and mental distress and life satisfaction are shown in figure 2. Higher social support (β=−0.086, 99% CI −0.113 to –0.060), more physical activity (β=−0.033, 99% CI −0.058 to –0.007), being actively working (β=−0.507, 99% CI −0.596 to –0.419) and higher relationship satisfaction (β=−0.174, 99% CI −0.199 to –0.149) were associated with lower symptoms of mental distress (see online supplemental table S12 in Appendix for crude and adjusted parameter estimates). Higher social support (β=0.104, 99% CI 0.080 to 0.127), being actively working (β=0.574, 99% CI 0.495 to 0.652) and higher relationship satisfaction (β=0.300, 99% CI 0.277 to 0.322) were associated with higher levels of life satisfaction (see online supplemental table S13 in Appendix for crude and adjusted parameter estimates).

### Associations before and during the pandemic

**Figure 3:**
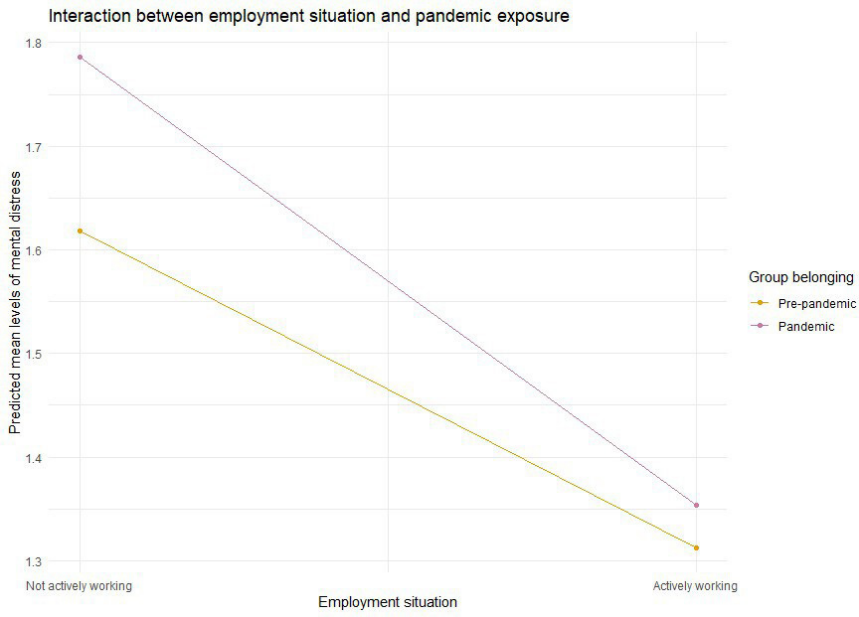
Interaction effect between employment situation and exposure to the pandemic on the association with mental distress.

With one exception, the associations between protective factors and outcomes did not differ between the pre-pandemic and pandemic groups. There was a significant interaction effect between employment situation and pandemic exposure on mental distress (β=−0.120, 99% CI −0.236 to –0.004, figure 2). This interaction effect is illustrated in figure 3. Being not actively working (either on sick leave or unemployed) was associated with higher levels of mental distress in the pandemic group compared with the pre-pandemic group.

### Sensitivity analyses

Robustness tests for relationship satisfaction showed similar results (see online supplemental tables S10 ans S11 in Appendix). Multiple regression models run on samples without item-level missing showed similar results (see online supplemental tables S14 and S15 in Appendix). Sensitivity with single item AUDIT-C showed similar results for life satisfaction, but a borderline significant result for mental distress (see online supplemental tables S16 and S17 in Appendix). The different results for mental distress and life satisfaction show the complexity when measuring alcohol consumption in this sample and indicate that mental distress and life satisfaction are related to the different aspects of alcohol consumption measured in AUDIT-C to different degrees. Using Q8 as a negative control did not show any significant interaction effects (see online supplemental tables S17 and S18 in Appendix).

## Discussion

### Summary of findings

This study used longitudinal data from more than 18 000 mothers to investigate whether the level of five protective factors—social support, physical activity, employment situation, alcohol consumption and relationship satisfaction—changed during the COVID-19 pandemic compared with pre-pandemic levels. Except for physical activity, the level of these factors was similar among all mothers. Associations were observed between the protective factors and both mental distress and life satisfaction, typically in opposite directions. Notably, the association between employment situation and mental distress was stronger in the pandemic group compared with the pre-pandemic group.

### Change in protective factors

Despite widespread disruptions to everyday life during the pandemic, most of the included protective factors showed a similar development from Q8 to Q14 among mothers in both groups, which suggests that these aspects of mothers’ lives are more robust to external stressors. Although the level of physical activity declined among all mothers from Q8 to Q14, the change over time was different among mothers in the pre-pandemic and pandemic groups. Specifically, the level of physical activity declined less over time for mothers in the pandemic group compared with the pre-pandemic group. This difference may reflect increased flexibility due to remote work or greater engagement in outdoor activities during the pandemic. While statistically significant, the actual impact on physical activity levels was minimal. The finding indicates that mothers in the pandemic group reported only 0.09 more mean hours of physical activity (a bit more than 5 min) from Q8 to Q14 compared with mothers in the pre-pandemic group. This small difference is unlikely to be of practical relevance at the individual level.

Contrary to a study on postpartum mothers reporting decreased social support during the pandemic,^20^ we did not find a pandemic effect among mothers with older children. This discrepancy may be due to differing concerns; having a newborn baby during the pandemic might have made mothers more isolated due to the fear of being infected or infecting the baby. In contrast, such fears might be lower in mothers with older children. Additionally, including baseline measures in our analyses strengthens their validity compared with studies relying solely on retrospective reports.

The observed similar level of employment situation in the pandemic and pre-pandemic groups over time contrasts findings from an Australian birth cohort study, where job loss and anticipated job loss were twice as common during the pandemic than before the pandemic.^19^ The different findings could be attributed to a different labour market in Norway, or selection bias in the MoBa sample.^42^ Also, the response range in Q14 covered a wide year span (between 2017 and 2023), compared with the Australian study, covering only the early stage of the pandemic (April–May 2020).

A lack of pandemic changes on alcohol use and relationship satisfaction has previously been reported.^22 24^ The null findings across many of the protective factors in our study might be due to a lack of financial strain, as greater financial strain during the pandemic has previously been shown to be associated with poorer relationship satisfaction over time.^43^ Most mothers in our study were actively working and might therefore not have experienced any economic uncertainties.

### Associations between protective factors, mental distress and life satisfaction

Three protective factors—social support, employment situation and relationship satisfaction—were associated with both mental distress and life satisfaction in mothers, aligning with previous studies.^19 25 27 28 30^ Physical activity was associated with mental distress, consistent with previous results.^28^ Contrary to our expectations, physical activity was not associated with life satisfaction, which has been previously documented.^29^ As SWLS measures global judgements of one’s life as a whole, mean hours of physical activity during a week may be more important for reducing acute symptoms of distress, as measured by SCL-8 using the past 2 weeks as reference period.

In our study, being actively working emerged as the strongest protective factor compared with other significant factors, with a 51%–57% change of a SD. The remaining significant factors demonstrated low to moderate effect sizes, with changes ranging from 3% to 30% of a SD. While these effects may appear small, they can still be clinically meaningful at the population level, particularly because such factors are modifiable through targeted interventions. Consequently, public health policies may aim to enhance social support, physical activity, employment opportunities and relationship satisfaction, as these factors have the potential to improve mental health and life satisfaction at the population level.

When investigating whether pandemic exposure moderated these associations, we found that the association between employment situation and mental distress was stronger within the pandemic group compared with the pre-pandemic group. As shown in figure 3, the protective effect of working corresponds to 0.31 lower SCL-8 score among mothers responding before the pandemic and 0.44 among mothers responding during the pandemic. This difference could be related to the uncertainty regarding the employment situation, especially during the early stages of the pandemic. Being temporarily or permanently laid off has serious consequences for household income, which could explain why being actively working was more important for the mental health of mothers during the pandemic compared with the pre-pandemic group of mothers. Being actively working during the pandemic could also indicate that the mothers were less isolated. While the clinical impact of this finding may be minimal on an individual level, even small reductions in symptoms of mental distress across a population could be important for public health efforts.

The associations of the other protective factors with mental health outcomes did not change according to pandemic exposure. This could indicate that the same protective factors are important both in normal times and during a global public health crisis like a pandemic, which also has implications when developing interventions aimed at promoting mental health and life satisfaction.

### Methodological considerations

Our study has some limitations. First, our results might be affected by self-report bias. Second, we applied a cross-sectional design when investigating associations, which limits the ability to address direction of causality. Third, there might be other confounding variables affecting the results that we have not measured. Fourth, the DID analyses rely on the parallel trends assumption, which cannot be empirically tested in our design due to having only two time points. However, because group assignment is determined solely by response timing and the pandemic represents an external, population-wide stressor, we consider the assumption plausible. Fifth, since Q14 data were collected over an extended period (2017–2023), classification into pre-pandemic and pandemic groups may partly capture secular trends unrelated to the COVID-19 pandemic. Our analyses therefore rely on the assumption that underlying time trends in protective factors and mental health outcomes would have evolved similarly in the absence of the pandemic. However, residual confounding by unmeasured time-varying factors cannot be excluded. Sixth, the low response rate, both initially (41%)^32^ and in Q14 (32%),^34^ raises concerns about generalisability due to non-response bias. For instance, studies have shown that the participants had a healthier lifestyle^44^ compared with the total population. Also, the negative pandemic effects might have been stronger on non-responders with lower education compared with responders (who are generally higher educated in MoBa).^42^ However, previous research suggests that non-response bias does not represent a strong threat to the validity of exposure-outcome associations.^44^ Lastly, the pandemic situation, restrictions and governmental support schemes in Norway might have differed from other countries, further limiting the generalisability. For instance, individuals who were temporarily laid off during the pandemic received financial support from the Norwegian Labour and Welfare Administration, which might have mitigated some of the negative effects of job loss. This context should be considered when interpreting the strength of the observed associations and their generalisability to settings with less comprehensive welfare systems.

## Conclusion

This study provides valuable insights into protective factors for maternal mental health and life satisfaction within the context of a welfare state. The similar levels of social support, employment situation, alcohol consumption and relationship satisfaction among all mothers over time suggest that these factors are robust to external stressors like a global pandemic. Importantly, these factors appear to play a consistently significant role in relation to mental distress and life satisfaction, both under ordinary circumstances and during public health crises. However, the heightened significance of employment status during the pandemic highlights its potential role as a buffer in times of economic uncertainty. Our findings may guide the development of targeted interventions to support maternal mental health and life satisfaction, by focusing on enhancing social support, physical activity, employment opportunities and relationship satisfaction. Future large-scale studies are needed to identify additional protective factors that may be particularly relevant during global crises. Future research should also explore potential causal pathways linking these factors to mental health and life satisfaction and investigate the mechanisms through which protective factors exert their influence.

## Supplementary material

10.1136/bmjopen-2025-110204 online supplemental file 1

## Data Availability

Data may be obtained from a third party and are not publicly available.
