# Supplementary material for: Protective factors for maternal mental health and life satisfaction during the COVID-19 pandemic: a longitudinal analysis
Source: BMJ Open. 2026 Jan 27;16(1):e110204. doi: 10.1136/bmjopen-2025-110204 (PMC12853534; doi:10.1136/bmjopen-2025-110204)
Supplement: online supplemental file 1 [file bmjopen-16-1-s001.docx]

Appendix

Protective factors for maternal mental health and life satisfaction during the COVID-19 pandemic: a longitudinal analysis

Pia Myklebust Johannessen^1,2^, Christian Madsen^3,4^, Rannveig Kaldager Hart^5,6^, Ingunn Olea Lund^1,2,7^, Johanne Hagen Pettersen^1,2^, Kristin Gustavson^2,7^, Espen Røysamb^8,7^, Ragnar Nesvåg^9^, Ragnhild Brandlistuen^7,1,8^ & Helga Ask^1,7,8^

^1^ PsychGen Center for Genetic Epidemiology and Mental Health, Norwegian Institute of Public Health, Oslo, Norway

^2^ Department of Psychology, University of Oslo, Oslo, Norway

^3^ Department of Disease Burden, Norwegian Institute of Public Health, Bergen, Norway

^4^ Centre for Disease Burden, Norwegian Institute of Public Health, Bergen, Norway

^5^ Department of Health Management and Health Economics, University of Oslo, Oslo, Norway

^6^ Centre for Fertility and Health, Norwegian Institute of Public Health, Oslo, Norway

^7^ Department of Child Health and Development, Norwegian Institute of Public Health, Oslo, Norway

^8^ PROMENTA Research Center, Department of Psychology, University of Oslo, Oslo, Norway

^9^ Division of Public Health and Prevention, Norwegian Institute of Public Health, Oslo, Norway

Corresponding author:

Pia Myklebust Johannessen

[Pia.jensen@fhi.no](mailto:Pia.jensen@fhi.no)

Table of content

[1. Deviations from preregistration 3](#_Toc201916037)

[2. Measures 5](#_Toc201916038)

[2.1 Mental health outcomes 5](#_Toc201916039)

[2.2 Protective factors 5](#_Toc201916040)

[3. Missing data in analytic samples 7](#_Toc201916041)

[4. Comparisons of analytic samples and MoBa full sample in Q1 8](#_Toc201916042)

[5. Statistical analyses 8](#_Toc201916043)

[5.1 Difference-in-Differences 8](#_Toc201916044)

[5.2 Regression Discontinuity Design (RDD) 9](#_Toc201916045)

[5.2.1 Assumptions 9](#_Toc201916046)

[5.1.2 Robustness tests RDD 9](#_Toc201916047)

[6. Change in protective factors before and after the onset of the COVID-19 pandemic 10](#_Toc201916048)

[6.1 Results social support 10](#_Toc201916049)

[6.2 Results physical activity 11](#_Toc201916050)

[6.3 Results employment situation 11](#_Toc201916051)

[6.4 Results alcohol consumption 12](#_Toc201916052)

[6.5 Results relationship satisfaction 13](#_Toc201916053)

[6.5.1 Results from sensitivity analyses for RDD 13](#_Toc201916054)

[7. Associations between protective factors, mental distress and life satisfaction 15](#_Toc201916055)

[7.1 Multiple regression analyses with mental distress as outcome 15](#_Toc201916056)

[7.2 Multiple regression analyses with life satisfaction as outcome 16](#_Toc201916057)

[7.3 Sensitivity analyses 17](#_Toc201916058)

[7.3.1 Without item-level missing 17](#_Toc201916059)

[7.3.2 Alcohol consumption sensitivity 19](#_Toc201916060)

[7.4 Robustness tests 21](#_Toc201916061)

[7.4.1. Mental distress 21](#_Toc201916062)

[7.4.2 Life satisfaction 22](#_Toc201916063)

[References 23](#_Toc201916064)

# 1. Deviations from preregistration

Table S1. Complete list of deviations from the preregistration

| Deviations | | | | |
| --- | --- | --- | --- | --- |
| Number | Details | Original wording | Description of deviation | Impact |
| #1 | Data exclusion | We will exclude mothers who only have answered one of the two required questionnaires (Q-8-years and Q-14-years) from our main sample. In some RD analysis, we will also include mothers who have answered Q-14-years only | We figured out that there was a delay in the data collection of Q14, resulting in some mothers answering Q14 when the child was older than 14-15 years. We therefore excluded mothers with children older than 15 during the first year of data collection in Q14 (2017). | This deviation was made to limit child age as a possible confounder in our analyses, and should strengthen the interpretation of our results. |
| #2 | Data exclusion | We will exclude mothers who only have answered one of the two required questionnaires (Q-8-years and Q-14-years) from our main sample. | We discovered that the answering distribution of Q8 was between the years 2001-2017. To make the answering distribution similar in Q8 and Q14, we excluded mothers who answered Q8 before 2011. | This deviation was made to make the baseline period and follow-up period more similar, and should strengthen the interpretation of our results. |
| #3 | Sample selection | We will only be using data from the Q-14-years | Due to the planned negative control with the Q8-data, this was a typing error. |  |
| #4 | Robustness tests | Based on our handling of missing data, we have two planned robustness tests. First, will repeat all analyses with only mothers who have filled out all items in a scale, and exclude those who have missing on one or more items in a scale. Second, we will do regression analyses where the outcome of interest is the number of complete scales. | We only performed the first robustness tests. | As the robustness test yielded similar results, we did not consider it meaningful to provide a second robustness test. |
| #5 | Specification of existing data | In the preregistration we specified the sample in Q14 to be 17566. | When starting the project, the data set got updated and increased to 26492 | This change should not influence the results. |
| #6 | Specification of pandemic groups | In the preregistration, the cut-off data separating the pre-pandemic group from the pandemic group was set to be March 12^th^ 2020 (date of Norwegian lockdown) for all analyses. | This was an error, and the cut-off date was changed to March 26^th^ 2020 for analyses using SCL-8 to account for the 2 weeks reference period. | This change should have no influence on the results, and strengthen the interpretation. |
| #7 | Choosing analyses | In the preregistration, we stated that we would use the RDD, but if the effects did not emerge sharply at the onset of the pandemic (March 12^th,^ 2020), we would use the dynamic DID or the simple DID if we lacked sufficient power. | We performed the simple DID on all protective factors except for a RDD on relationship satisfaction which lacked a baseline measure. | In a previous article, we found that the results from the RDD analyses were not robust. In addition, we found that the simple and dynamic DID yielded similar results with regards to interpreting a pandemic effect. Consequently, we decided to only use the simple DID in the present study in accordance with the principle of parsimony. |
| #8 | Covariates | Marital status | Data quality | Due to poor data quality, we decided not to include marital status as a covariate. It is possible that some of the observed associations are confounded by marital status. |

# 2. Information on variables

In the regression analyses, the outcomes were standardized before entered into the model using the scale() function in base R, and the predictors in the regression models were standardized by using the summ() function in jtools (Long, 2022).

## 2.1 Mental health outcomes

**Mental distress** was measured by SCL-8, which includes the following items from the SCL-25 (Hesbacher et al., 1980); 4 items measuring symptoms of anxiety (“Feeling fearful”, “Nervousness or shakiness inside”, “Feeling tense or keyed up” and “Suddenly scared for no reason”) and 4 items measuring symptoms of depression (“Feeling hopeless about the future”, “Feeling blue”, “Worrying too much about things” and “Feeling everything is an effort”). SCL-8 showed a good reliability, with a Cronbach’s alpha of .88 and .89 in the two waves of data collection. **Life satisfaction** was measured by SWLS, which consists of five items measuring global life satisfaction. In the present study, SWLS showed good reliability, with a Cronbach’s alpha of .91 and .92 in the two waves of data collection.

## 2.2 Protective factors

**Social support** was measured by two MoBa specific questions. The first was phrased *‘Do you have anyone other than your husband/partner you can ask for advice in a difficult situation?’*, with the response options 1 (No), 2 (Yes, 1-2 people) and 3 (Yes, more than 2 people). The second item was phrased *‘How often do you meet or talk on the telephone with your family (other than your husband/partner and children) or close friends?’*, with the response options: 1 (Once a month or less), 2 (Two to three times a month), and 3 (More than twice a week). Operationalizing social support as mean of the responses to the two questions was inspired by previous work (Magnus et al., 2018).

**Physical activity** was measured by three questions referring to the frequency of physical activity leading the mother to get out of breath or sweat (adapted from Sagatun et al., 2007): «How often do you exercise for up to 30 minutes”, “How often do you exercise for 30-60 minutes” and “How often do you exercise for more than 60 minutes”. Each question had the response options: “never”, “less than once a week”, “once a week”, “2 times a week”, “3-4 times a week”, and “5 times a week or more”. As a first step, we converted the response options into the frequency of physical activity during a week. The response options were recoded as: 0 (“never”), 0.5 (“less than once a week), 1 (“once a week”), 2 (“two times a week”), 3.5 (“3-4 times a week”) and 5 (“5 times a week or more”). Next, we created three subscales to calculate the number of hours of exercising up to 30 minutes, for 30-60 minutes and more than 60 minutes. The subscale physical activity for 30 minutes was calculated by multiplying the first question with 0,5 hours. The subscale physical activity for 30-60 minutes was calculated by multiplying the second question with 0.75 hours, as 45 minutes is between 30-60 minutes. The last subscale physical activity for more than 60 minutes was calculated by multiplying the third question with 1 hour. The complete scale was calculated as a mean score, by summing the subscales divided by the number of answered subscales. Individuals were included in the analyses if they had filled in one of the three subscales of physical activity. We operationalized physical activity as a continuous variable by summing three non-overlapping weekly duration categories and dividing by number of answered items, to calculate the mean hours of moderate-to-vigorous physical activity per week. This approach assumes an approximately linear and monotonic relationship between weekly exercise duration and mental health outcomes, an assumption widely adopted in epidemiological research. Treating physical activity as continuous facilitates interpretability and comparability across studies.

**Employment situation** was measured by the question “Are you currently in paid employment?”. In Q-8-years, the response options were: “Yes”, “Yes, but I am on partial sick leave”, “Yes, but I am on full sick leave” and “No”. In Q-14-years, the response options were: “Yes, full time work”, “Yes, part time, “Yes, but am currently on sick leave” and “No”.

**Alcohol consumption** was measured by AUDIT-C. The response options in the two first items deviated from the original scale. To the first question “How often do you drink alcohol now?”, the categories in MoBa were: “Never”, “Less than once a month”, “About 1-3 times per month”, “About once per week”, “About 2-3 times per week”, “About 4-5 times per week” and “About 6-7 times per week”. To the second question “How many alcohol units do you have on a typical day when you are drinking?”, the categories in MoBa were: “Less than 1”, “1-2”, “3-4”, “5-6”, “7-9” and “10 or more”. In the present study, AUDIT-C showed a poor reliability in both Q-8-years and Q-14-years, with a Cronbach’s alpha of .35 and .47 respectively.

**Relationship satisfaction**: Participants who have a husband/boyfriend/partner were asked to indicate to what extent they agree with the descriptions, such as “I am very happy with our relationship”. In the present study, RS5 showed a god reliability, with a Cronbach’s alpha of .91.

# 3. Missing data in analytic samples

On predictors and outcomes, missing refers to individuals who have more than 50% missing on items within each scale (specific details are described in article and results are presented in Table S2). On covariates, missing refers to those we do not have any information about. Dataset 1 is a panel data set in long format, therefore missing is calculated as the sum of missing from both Q8 and Q14. In Dataset 2, missing on the predictors and outcomes are only from Q14.

Table S2. Missing data in the two Datasets

|  | Missing | |
| --- | --- | --- |
| Variables | Dataset 1  (n= 36,030) | Dataset 2  (n=18,339) |
| SCL-8 | - | 68  (0,4%) |
| SWLS | - | 79  (0,4%) |
| RS-scale (Q14 only) | 1714  (4,8%) | 1732^a^  (9,4%) |
| AUDIT-C | 271  (0,8%) | 46  (0,3%) |
| Social support | 442  (1,2%) | 63  (0,3%) |
| Physical activity | 430  (1,2%) | 57  (0,3%) |
| Working situation | 326  (0,9%) | 69  (0,4%) |
| Age Q14 | 120  (0,3%) | 60  (0,3%) |
| Age Q8 | 420  (1,2%) | 211  (1,2%) |
| Education | 22  (0,1%) | 12  (0,1%) |
| Living situation Q8 | 532  (1,5%) | 273  (1,5%) |

Notes: SCL-8; Hopkins Symptom Checklist-8, SWLS; The Satisfaction With Life Scale, RS-scale; The Relationship Satisfaction Scale, AUDIT-C; the Alcohol Use Disorders Identification Test-Consumption.

^a^ Individuals are included in the scale with information on at least one item

# 4. Comparisons of analytic samples and MoBa full sample in Q1

Table S3. Comparison of analytic samples and full MoBa sample in Q1

|  |  | Dataset 1  (n= 18 015) | Dataset 2  (n= 18 339) | MoBa sample  (n = 101 565) |
| --- | --- | --- | --- | --- |
| Education  n(%) | 9-year secondary school | 177  (1.0%) | 183  (1.0%) | 2745  (2.7%) |
|  | 1-2 year high school | 434  (2.4%) | 451  (2.5%) | 4 934  (4.9%) |
|  | Vocational high school | 1 537  (8.5%) | 1 560  (8.5%) | 12 542  (12.3%) |
|  | 3-year high school general studies, junior college | 2 047  (11.4%) | 2 071  (11.3%) | 14 252  (14.0%) |
|  | Regional technical college, 4-year university degree | 8 108  (45.0%) | 8 267  (45.1%) | 39 354  (38.7%) |
|  | University, technical college, more than 4 years | 4 763  (26.4%) | 4 841  (26.4%) | 22 535  (22.2%) |
| Mean SCL-5 (SD) |  | 1.22  (0.36) | 1.22  (0.36) | 1.25  (0.39) |
| Mean SWLS (SD) |  | 5.75  (0.99) | 5.76  (0.99) | 5.67  (1.07) |

# 5. Statistical analyses

## 5.1 Difference-in-Differences

The Difference-in-Differences (DID) model was estimated with the following form:

$$Y_{it}=\beta_{0}+\beta_{1}{Group}_{it}+\beta_{2}{Time}_{it}+\beta_{3}({Group}_{it}\times{Time}_{it})+\boldsymbol{\beta}_{\boldsymbol{4}}\boldsymbol{X+}\varepsilon_{it}$$

Y represents the outcome of interest (i.e., protective factors), Group is a dummy variable indicating whether the mothers belong to the pre-pandemic group (coded as 0) or the pandemic group (coded as 1). Time is a dummy variable distinguishing the baseline period (Q8; coded as 0) from the follow-up period (Q14; coded as 1). The coefficient of interest is, *β_3_*, captures the interaction effect between Group and Time.

## 5.2 Regression Discontinuity Design (RDD)

The egression Discontinuity Design (RDD) model was estimated with the following regression equation:

$$Y_{i}=\beta_{0}+\beta_{1}Group+{\beta_{2}Answer_{time}}_{i}+ \boldsymbol{\beta}_{\boldsymbol{3}}\boldsymbol{X}+ \varepsilon_{i}$$

Y represents the average outcome of interest (relationship satisfaction), while Group is a dummy variable separating the pre-pandemic group (coded as 0) from the pandemic group (coded as 1). Answer time is the running variable and represent the date mothers filled out the Q-14-year questionnaire. In our model, March 12, 2020, was used as the cut-off date. The running variable was centered on March 12, 2020, counting months before and after this date.

### 5.2.1 Assumptions

To investigate whether the running variable was continuous at cut off, which is an assumption in RDD, we performed the Density test (McCrary, 2008) (see Figure S1). In our study, time centered (running variable) was not continuous around the cut-off. Since the time when answering the Q14 is decided by the date of the child’s birth, participants cannot manipulate whether they received Q14 before or after the lockdown. Therefore, we assume the assumption still holds.


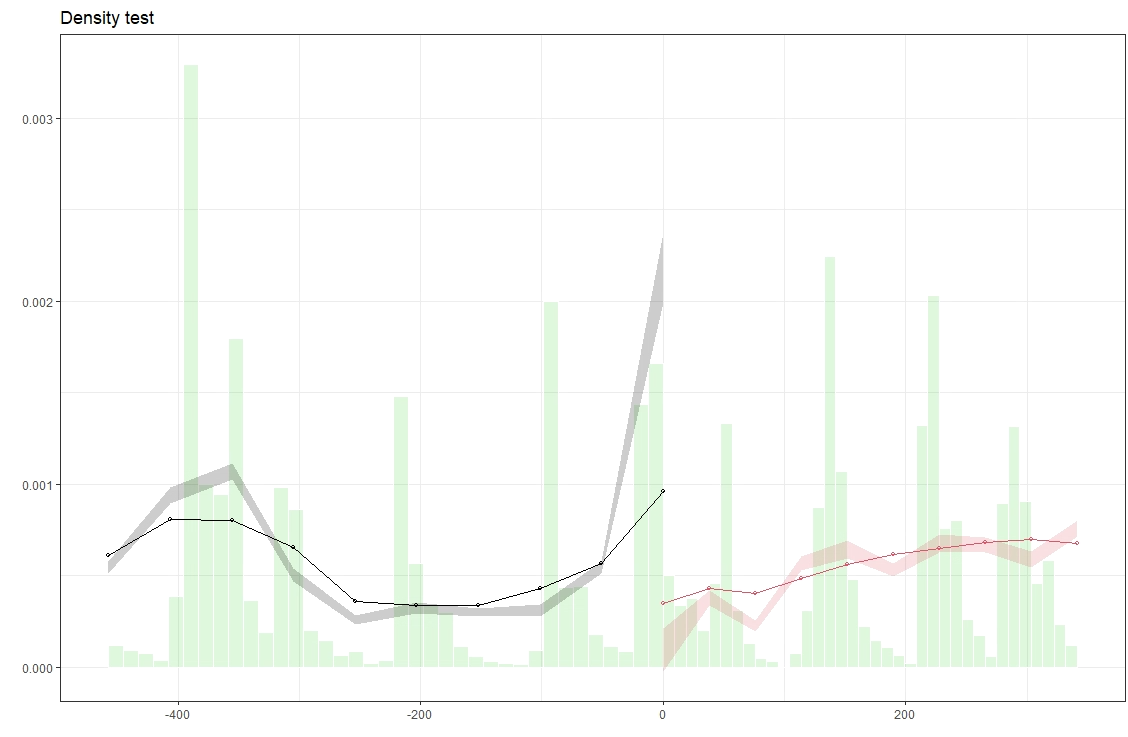


Figure S1. Density test of running variable

### 5.1.2 Robustness tests RDD

To check whether the findings were robust, we conducted sensitivity analyses including the use of placebo cut-offs, varying bandwidth, non-linear trend assessments, and a non-parametric estimation. Placebo cut-offs will only be performed if the main result is significant, and involves setting alternative cut-off points on the running variable. In the whole sample March 12, 2019 will be used as a placebo cut-off, and in a pre-pandemic sub-sample March 12, 2018 will be used as a placebo cut-off to determine whether results were specific to the onset of the Norwegian lockdown. Significant placebo cut-offs would indicate that some other factors than the COVID-19 pandemic affect the results. To vary the bandwidth, we used a data-driven method aimed at minimizing Mean Squared Error (MSE) (Imbens & Kalyanaraman, 2012) by using the rdbwselect function in rdrobust() (Calonico et al., 2023). Curvilinear trends were also examined to ensure the robustness of the findings and to test whether this improved model fit. The final robustness test was applying a non-parametric estimation of the pandemic effect at lockdown by using the rdrobust() package in R (Calonico et al., 2023). This function integrates robust standard errors, bias correction and MSE-optimal bandwidths to produce robust bias corrected RD confidence intervals (Calonico et al., 2014).

# 6. Change in protective factors before and after the onset of the COVID-19 pandemic

## 6.1 Results social support

**Table S4.** Simple difference-in-differences estimates of the pandemic effect on social support among Norwegian mothers, by model specification.

|  | Unadjusted model | | | Adjusted model^a^ | | |
| --- | --- | --- | --- | --- | --- | --- |
|  | Estimate  (S.E) | 99% CI | P-value | Estimate  (S.E) | 99% CI | P-value |
| Group | -.020  (.007) | -.037 - -.002 | .0039 | -.019  (.007) | -.037 - -.001 | .0064 |
| Time | -.091  (.005) | -.104 - -.077 | <.001 | -.092  (.005) | -.106 - -.078 | <.001 |
| Group x Time | -.016  (.007) | -.034 - .003 | .0286 | -.013  (.007) | -.032 - .005 | .0634 |
| Constant | 2.578  (.005) | 2.565 – 2.591 | <.001 | 2.739  (.053) | 2.603 – 2.875 | <.001 |
| Adjusted R^2^ | .012 |  |  | .019 |  |  |
| Observations | 35 588 |  |  | 34 859 |  |  |

Note: Standard errors are clustered at individual level.

^a^Adjusted for age, education and living situation

## 6.2 Results physical activity

**Table S5.** Simple difference-in-differences estimates of the pandemic effect on physical activity among Norwegian mothers, by model specification.

|  | Unadjusted model | | | Adjusted model^a^ | | |
| --- | --- | --- | --- | --- | --- | --- |
|  | Estimate  (S.E) | 99% CI | P-value | Estimate  (S.E) | 99% CI | P-value |
| Group | -.061  (.015) | -.099 – -.023 | <.001 | -.057  (.015) | -.096 – -.019 | .0001 |
| Time | -.283  (.012) | -.313 – -.253 | <.001 | -.281  (.012) | -.311 – -.251 | <.001 |
| Group x Time | .087  (.015) | .048 - .126 | <.001 | .085  (.015) | .046 - .124 | <.001 |
| Constant | 1.411  (.011) | 1.382 – 1.440 | <.001 | 1.523  (.100) | 1.265 – 1.780 | <.001 |
| Adjusted R^2^ | .017 |  |  | .018 |  |  |
| Observations | 35 600 |  |  | 34 912 |  |  |

Note: Standard errors are clustered at individual level.

^a^Adjusted for age, education and living situation

## 6.3 Results employment situation

**Table S6.** Simple difference-in-differences estimates of the pandemic effect on sick leave among Norwegian mothers, by model specification.

|  | Unadjusted model | | | Adjusted model^a^ | | |
| --- | --- | --- | --- | --- | --- | --- |
|  | Odds ratio (S.E) | 99% CI | P-value | Odds Ratio  (S.E) | 99% CI | P-value |
| Group | .977  (.066) | .824 – 1.158 | .7243 | 1.060  (.068) | .891 – 1.262 | .3874 |
| Time | .686  (.076) | .564 -.835 | <.001 | .682  (.078) | .558 - .833 | <.001 |
| Group x Time | 1.093  (.099) | .847 – 1.411 | .3669 | 1.085  (.101) | .837 – 1.408 | .4165 |
| Constant | .064  (.050) | .056 – .073 | <.001 | .475  (.457) | .147 – 1.540 | .1029 |
| Observations | 33 775 |  |  | 33 083 |  |  |

Note: Standard errors are clustered at individual level.

^a^Adjusted for age, education and living situation

**Table S7.** Simple difference-in-differences estimates of the pandemic effect on unemployment among Norwegian mothers, by model specification.

|  | Unadjusted model | | | Adjusted model^a^ | | |
| --- | --- | --- | --- | --- | --- | --- |
|  | Odds Ratio  (S.E.) | 99% CI | P-value | Odds Ratio  (S.E) | 99% CI | P-value |
| Group | 1.000  (.067) | .844 – 1.186 | .9967 | 1.141 (.069) | .955 – 1.364 | .0561 |
| Time | 1.013  (.059) | .832 – 1.232 | .8326 | 1.000  (.061) | .855 – 1.169 | .9989 |
| Group x Time | .937  (.078) | .726 – 1.208 | .3998 | .950  (.080) | .774 – 1.167 | .5215 |
| Constant | .061  (.050) | .054 - .069 | <.001 | 2.462 (.474) | .725 – 8.354 | .0576 |
| Observations | 33 966 |  |  | 33 285 |  |  |

Note: Standard errors are clustered at individual level.

^a^Adjusted for age, education and living situation

## 6.4 Results alcohol consumption

**Table S8.** Simple difference-in-differences estimates of the pandemic effect on alcohol consumption among Norwegian mothers, by model specification.

|  | Unadjusted model | | | Adjusted model^a^ | | |
| --- | --- | --- | --- | --- | --- | --- |
|  | Estimate  (S.E.) | 99% CI | P-value | Estimate  (S.E.) | 99% CI | P-value |
| Group | .021  (.008) | .002 - .040 | .0051 | .015  (.008) | -.005 - .035 | .0468 |
| Time | .013  (.005) | .001 - .025 | .0039 | .013  (.005) | .001 - .025 | .0057 |
| Group x Time | .011  (.006) | -.005 - .027 | .0716 | .011  (.006) | -.004 - .028 | .0565 |
| Constant | .843  (.006) | .828 - .858 | <.001 | .872  (.064) | .709 – 1.036 | <.001 |
| Adjusted R^2^ | .001 |  |  | .004 |  |  |
| Observations | 35 759 |  |  | 35 022 |  |  |

Note: Standard errors are clustered at individual level.

^a^Adjusted for age, education and living situation

## 6.5 Results relationship satisfaction

**Table S9.** Regression discontinuity estimates of the effect of the Norwegian lockdown on relationship satisfaction among Norwegian mothers, by model specification

|  | Unadjusted model | | | Adjusted model^a^ | | |
| --- | --- | --- | --- | --- | --- | --- |
|  | Estimate  (S.E.) | 99% CI | p-value | Estimate  (S.E.) | 99% CI | p-value |
| Group | -.016  (.029) | -.091 - .058 | .5712 | -.010  (.029) | -.086 - .065 | .7238 |
| Time_centered | .000  (.001) | -.002 - .002 | .5751 | .001  (001) | -.001 - .003 | .1874 |
| Constant | 4.780  (.016) | 4.738 – 4.822 | <.001 | 5.375  (.121) | 5.065 – 5.686 | <.001 |
| Adjusted R^2^ | -.000 |  |  | .008 |  |  |
| Observations | 16 301 |  |  | 15 914 |  |  |

Note: Standard errors are estimated with heteroskedasticity-robust standard errors

^a^Adjusted for age, education and living situation

### 6.5.1 Results from sensitivity analyses for RDD

**Table S10.** Robustness results for relationship satisfaction using optimal bandwidth in regression discontinuity design

|  | Adjusted model with optimal bandwidth^a,b^ | | |
| --- | --- | --- | --- |
|  | Estimate  (S.E.) | 99% CI | p-value |
| Group | -.123  (.056) | -.267 - .021 | .0275 |
| Time_centered | .013  (.006) | -.003- .029 | .0384 |
| Time_centered^2^ | .001  (.001) | -.001 - .004 | .1027 |
| Constant | 5.587  (.244) | 4.957 – 6.216 | <.001 |
| Adjusted R^2^ | .010 |  |  |
| Observations | 4 010 |  |  |

Note: Standard errors are estimated with heteroskedasticity-robust standard errors

^a^ Bandwidth of 7.649

^b^Adjusted for age, education and living situation

**Table S11**. Non-parametric robustness results for relationship satisfaction using regression discontinuity design

|  | Non-parametric unadjusted | | | Non-parametric adjusted^a^ | | |
| --- | --- | --- | --- | --- | --- | --- |
|  | Estimate  (S.E.) | 99% CI | P-value | Estimate  (S.E.) | 99% CI | P-value |
| Conventional | -.112  (.075) | -.259 - .034 | .134 | -.102  (.075) | -.250 - .046 | .176 |
| Robust | -.133  (.100) | -329 - .063 | .182 | -.124  (.101) | -.322 - .073 | .217 |
| Observations | 4 104 |  |  | 4 089 |  |  |
| Banwidth | 7.649 |  |  | 7.595 |  |  |

^a^Adjusted for age, education and living situation

# 7. Associations between protective factors, mental distress and life satisfaction

## We assessed multicollinearity among protective factors prior to the regression analyses. Variance inflation factors (VIFs) ranged from 1 to 3 (with response date and pandemic group at the upper end), and pairwise correlations were low (r = 0.01-0.09), well below commonly used thresholds, indicating no problematic multicollinearity

## 7.1 Multiple regression analyses with mental distress as outcome

**Table S12**. Regression models for change in mental distress predicted by protective factors and their interaction with pandemic group belonging

|  | Crude models | | | | Adjusted model^a^ | | | |
| --- | --- | --- | --- | --- | --- | --- | --- | --- |
|  | β  (S.E.) | 99% CI | p-value | R^2^ | β  (S.E.) | 99% CI | p-value | R^2^ |
| Social support | -.204 (.007) | -.222– -.185 | <.001 | .041 | -.086  (.010) | -.113 - -.060 | <.001 | .317 |
| Physical activity | -.080  (.007) | -.099 – -.061 | <.001 | .006 | -.033  (.010) | -.058 – -.007 | .0010 |  |
| Employment situation | -.814  (.025) | -.877 – -.750 | <.001 | .057 | -.507 (.034) | -.596 – -.419 | <.001 |  |
| Alcohol consumption | -.006 (.007) | -.025 - .013 | .4481 | -.000 | .020  (.010) | -.006 - .046 | .0441 |  |
| Relationship satisfaction | -.287  (.007) | -.306 – -.269 | <.001 | .087 | -.174  (.010) | -.199 – -.149 | <.001 |  |
| Baseline SCL-8 |  |  |  |  | .389  (.007) | .372 - .406 | <.001 |  |
| Time |  |  |  |  | .062  (.012) | .032 - .092 | <.001 |  |
| Pandemic group |  |  |  |  | .095 (.047) | -.027 - .217 | .0440 |  |
| Social support x Pandemic group |  |  |  |  | .013  (.013) | -.047 - .021 | .3337 |  |
| Physical activity x Pandemic group |  |  |  |  | -.008 (.013) | -.042 - .025 | .5120 |  |
| Employment situation x Pandemic group |  |  |  |  | -.120  (.045) | -.236 – -.004 | .0076 |  |
| Alcohol consumption x Pandemic group |  |  |  |  | -.012 (.013) | -.045 - .022 | .3617 |  |
| Relationship satisfaction x Pandemic group |  |  |  |  | -.019 (.013) | -.052 - .014 | .1452 |  |

Note: Coefficients are standardized

^a^Adjusted for age and education

## 7.2 Multiple regression analyses with life satisfaction as outcome

**Table S13.** Regression models for change in life satisfaction predicted by protective factors and their interaction with pandemic group belonging

|  | Crude models | | | | Adjusted model | | | |
| --- | --- | --- | --- | --- | --- | --- | --- | --- |
|  | β  (S.E.) | 99% CI | p-value | R^2^ | β  (S.E.) | 99% CI | p-value | R^2^ |
| Social support | .234  (.007) | .216 - .253 | <.001 | .055 | .104  (.009) | .080 - .127 | <.001 | .435 |
| Physical activity | .079  (.007) | .060 - .098 | <.001 | .006 | .023  (.009) | -.000 - .045 | .0107 |  |
| Employment situation | .867 (.024) | .804 - .929 | <.001 | .064 | .574  (.031) | .495 - .652 | <.001 |  |
| Alcohol consumption | .052  (.007) | .033 - .071 | <.001 | .003 | .011  (.009) | -.012 - .034 | .2211 |  |
| Relationship satisfaction | .445  (.007) | .429 - .462 | <.001 | .217 | .300  (.009) | .277 - .322 | <.001 |  |
| Baseline SWLS |  |  |  |  | .361  (.006) | .345 - .377 | <.001 |  |
| Time |  |  |  |  | -.046  (.010) | -.073 – -.020 | <.001 |  |
| Pandemic group |  |  |  |  | .033  (.042) | -.075 - .142 | .4286 |  |
| Social support x Pandemic group |  |  |  |  | -.001  (.012) | -.031 - .030 | .9632 |  |
| Physical activity x Pandemic group |  |  |  |  | -.004  (.012) | -.033 - .026 | .7602 |  |
| Employment situation x Pandemic group |  |  |  |  | .025  (.040) | -.078 - .129 | .5300 |  |
| Alcohol consumption x Pandemic group |  |  |  |  | .007  (.012) | -.023 - .036 | .5738 |  |
| Relationship satisfaction x Pandemic group |  |  |  |  | .010  (.012) | -.020 - .040 | .3758 |  |

Note: Coefficients are standardized

^a^Adjusted for age and education

## 7.3 Sensitivity analyses

### 7.3.1 Without item-level missing

**Table S14.** Sensitivity analysis for the association between protective factors and mental distress in a sample without missing on SCL-8

|  | Adjusted model^a^ | | | |
| --- | --- | --- | --- | --- |
|  | β  (S.E.) | 99% CI | p-value | R2 |
| Social support | -.089 (.010) | -.116 - -.063 | <.001 | .320 |
| Physical activity | -.032 (.010) | -.058 – -.007 | .0012 |  |
| Employment situation | -.510 (.035) | -.599 – -.421 | <.001 |  |
| Alcohol consumption | .021 (.010) | -.005 - .047 | .0349 |  |
| Relationship satisfaction | -.175  (.010) | -.201 – -.150 | <.001 |  |
| Baseline SCL-8 | .391  (.007) | .373 - .408 | <.001 |  |
| Time | .062  (.012) | .032 - .092 | <.001 |  |
| Pandemic group | .098  (.048) | -.025 - .221 | .0408 |  |
| Social support x Pandemic group | .011 (.013) | -.045 - .023 | .4053 |  |
| Physical activity x Pandemic group | -.008 (.013) | -.041 - .026 | .5648 |  |
| Employment situation x Pandemic group | -.123  (.045) | -.240 – -.006 | .0068 |  |
| Alcohol consumption x Pandemic group | -.011  (.013) | -.045 - .022 | .3889 |  |
| Relationship satisfaction x Pandemic group | -.017 (.013) | -.051 - .016 | .1885 |  |

Note: Coefficients are standardized

^a^Adjusted for age and education

**Table S15.** Sensitivity analysis for the association between protective factors and life satisfaction in a sample without missing on SWLS

|  | Adjusted model^a^ | | | |
| --- | --- | --- | --- | --- |
|  | β  (S.E.) | 99% CI | p-value | R2 |
| Social support | .105  (.009) | .082 - .129 | <.001 | .436 |
| Physical activity | .021  (.009) | -.002 - .044 | .0171 |  |
| Employment situation | .571  (.031) | .492- .651 | <.001 |  |
| Alcohol consumption | .010  (.009) | -.013 - .033 | .2720 |  |
| Relationship satisfaction | .299  (.009) | .276 - .322 | <.001 |  |
| Baseline SWLS | .361 (.006) | .345 - .377 | <.001 |  |
| Time | -.045  (.010) | -.072 – -.019 | <.001 |  |
| Pandemic group | .028 (.043) | -.082 - .137 | .5120 |  |
| Social support x Pandemic group | -.002  (.012) | -.033 - .028 | .8513 |  |
| Physical activity x Pandemic group | -.001 (.012) | -.030 - .029 | .9643 |  |
| Employment situation x Pandemic group | .027 (.040) | -.077 - .131 | .5014 |  |
| Alcohol consumption x Pandemic group | .008  (.012) | -.022 - .038 | .4812 |  |
| Relationship satisfaction x Pandemic group | 011 (.012) | -.019 - .041 | .3308 |  |

Note: Coefficients are standardized

^a^Adjusted for age and education

### 7.3.2 Alcohol consumption sensitivity

**Table S16**. Alcohol consumption sensitivity analysis for the association between protective factors and mental distress

|  | Adjusted model^a^ | | | |
| --- | --- | --- | --- | --- |
|  | β  (S.E.) | 99% CI | p-value | R2 |
| Social support | -.086  (.010) | -.112 - -.060 | <.001 | .317 |
| Physical activity | -.033  (.010) | -.058 – -.007 | .0009 |  |
| Employment situation | -.509  (.034) | -.597 – -.421 | <.001 |  |
| Frequency of alcohol consumption | .027  (.010) | .001 - .054 | .0069 |  |
| Relationship satisfaction | -.175  (.010) | -.200 – -.150 | <.001 |  |
| Baseline SCL-8 | .389  (.007) | .372- .406 | <.001 |  |
| Time | .063  (.012) | .033 - .093 | <.001 |  |
| Pandemic group | .092  (.047) | -.030 - .214 | .0512 |  |
| Social support x Pandemic group | .013  (.013) | -.047 - .021 | .3281 |  |
| Physical activity x Pandemic group | -.008  (.013) | -.042 - .025 | .5147 |  |
| Employment situation x Pandemic group | -.117  (.045) | -.233 – -.001 | .0091 |  |
| Frequency of alcohol consumption x Pandemic group | -.019  (.013) | -.053 - .015 | .1461 |  |
| Relationship satisfaction x Pandemic group | -.018  (.013) | -.052 - .015 | .1557 |  |

Note: Coefficients are standardized

^a^Adjusted for age and education

**Table S17.** Alcohol consumption sensitivity analysis for the association between protective factors and life satisfaction

|  | Adjusted model^a^ | | | |
| --- | --- | --- | --- | --- |
|  | β  (S.E.) | 99% CI | p-value | R2 |
| Social support | .104  (.009) | .080 - .127 | <.001 | .435 |
| Physical activity | .022 (.009) | -.000 - .045 | .0116 |  |
| Employment situation | .573 (.031) | .495 - .652 | <.001 |  |
| Frequency of alcohol consumption | .016 (.009) | -.008 - .039 | .0872 |  |
| Relationship satisfaction | .299  (.009) | .277 - .322 | <.001 |  |
| Baseline SWLS | .360 (.006) | .344 - .376 | <.001 |  |
| Time | -.045  (.010) | -.071 – -.018 | <.001 |  |
| Pandemic group | .030 (.042) | -.079 - .138 | .4826 |  |
| Social support x Pandemic group | .000  (.012) | -.030 - .030 | .9958 |  |
| Physical activity x Pandemic group | -.003  (.012) | -.033 - .027 | .7870 |  |
| Employment situation x Pandemic group | .026  (.040) | -.077 - .130 | .5154 |  |
| Frequency of alcohol consumption x Pandemic group | .001 (.012) | -.029 - .031 | .9012 |  |
| Relationship satisfaction x Pandemic group | .010 (.012) | -.020 - .040 | .3792 |  |

Note: Coefficients are standardized

^a^Adjusted for age and education

## 7.4 Robustness tests

### 7.4.1. Mental distress

**Table S18.** Robustness test investigating the association between protective factors and mental distress with Q-8-years as a negative control.

|  | Adjusted model^a^ | | | |
| --- | --- | --- | --- | --- |
|  | β  (S.E.) | 99% CI | p-value | R2 |
| Social support | -.188  (.011) | -.216 – -.159 | <.001 | .102 |
| Physical activity | -.046  (.011) | -.073 – -.018 | <.001 |  |
| Employment situation | -.758 (.035) | -.847 – -.668 | <.001 |  |
| Alcohol consumption | .058 (.011) | .030 - .086 | <.001 |  |
| Pandemic group | .044 (.043) | -.068 - .155 | .3132 |  |
| Social support x Pandemic group | .015 (.015) | -.052 - .022 | .2938 |  |
| Physical activity x Pandemic group | -.015 (.014) | -.052- .022 | .2961 |  |
| Employment situation x Pandemic group | .004 (.046) | -.114 - .122 | .9359 |  |
| Alcohol consumption x Pandemic group | .004 (.014) | -.033 - .041 | .7637 |  |

Note: Coefficients are standardized

^a^Adjusted for age and education

### 7.4.2 Life satisfaction

**Table S19.** Robustness test investigating the association between protective factors and life satisfaction with Q-8-years as a negative control.

|  | Adjusted model^a^ | | | |
| --- | --- | --- | --- | --- |
|  | β  (S.E.) | 99% CI | p-value | R2 |
| Social support | .208  (.012) | .178 - .238 | <.001 | .107 |
| Physical activity | .064  (.011) | .035 - .092 | <.001 |  |
| Employment situation | .608 (.036) | .515 - .701 | <.001 |  |
| Alcohol consumption | -.052  (.011) | -.081 - -.023 | <.001 |  |
| Pandemic group | -.069  (.045) | -.184 - .046 | .1244 |  |
| Social support x Pandemic group | .016  (.015) | -.022 - .055 | .2756 |  |
| Physical activity x Pandemic group | -.010  (.015) | -.048 - .028 | .4921 |  |
| Employment situation x Pandemic group | .079  (.047) | -.043 - .201 | .0952 |  |
| Alcohol consumption x Pandemic group | .016  (.015) | -.023 - .054 | .2894 |  |

Note: Coefficients are standardized

^a^Adjusted for age and education

# References

Calonico, S., Cattaneo, M. D., Farrell, M., & Titiunik, R. (2023). *rdrobust: Robust Data-Driven Statistical Inference in Regression-Discontinuity Designs*. In (Version R package version 2.2) <https://CRAN.R-project.org/package=rdrobust>

Calonico, S., Cattaneo, M. D., & Titiunik, R. (2014). Robust nonparametric confidence intervals for regression‐discontinuity designs. *Econometrica*, *82*(6), 2295-2326.

Hesbacher, P. T., Rickels, K., Morris, R. J., Newman, H., & Rosenfeld, H. (1980). Psychiatric illness in family practice. *The Journal of Clinical Psychiatry*, *41*(1), 6-10.

Imbens, G., & Kalyanaraman, K. (2012). Optimal bandwidth choice for the regression discontinuity estimator. *The Review of economic studies*, *79*(3), 933-959. <https://doi.org/https://doi.org/10.1093/restud/rdr043>

Long, J. (2022). *jtools: Analysis and Presentation of Social Scientific Data. R package version 2.2.0*. In <https://cran.r-project.org/package=jtools>

Magnus, M. C., Wright, R. J., Røysamb, E., Parr, C. L., Karlstad, Ø., Page, C. M., Nafstad, P., Håberg, S. E., London, S. J., & Nystad, W. (2018). Association of maternal psychosocial stress with increased risk of asthma development in offspring. *American journal of epidemiology*, *187*(6), 1199-1209. <https://doi.org/https://doi.org/10.1093/aje/kwx366>

McCrary, J. (2008). Manipulation of the running variable in the regression discontinuity design: A density test. *Journal of Econometrics*, *142*(2), 698-714. <https://doi.org/https://doi.org/10.1016/j.jeconom.2007.05.005>

Sagatun, A., Søgaard, A. J., Bjertness, E., Selmer, R., & Heyerdahl, S. (2007). The association between weekly hours of physical activity and mental health: a three-year follow-up study of 15–16-year-old students in the city of Oslo, Norway. *BMC Public Health*, *7*, 1-9, Article 155. <https://doi.org/https://doi.org/10.1186/1471-2458-7-155>
