# Supplementary material for: Protective factors for maternal mental health and life satisfaction during the COVID-19 pandemic: a longitudinal analysis
Source: BMJ Open. 2026 Jan 27;16(1):e110204. doi: 10.1136/bmjopen-2025-110204 (PMC12853534; doi:10.1136/bmjopen-2025-110204)
Supplement: Supplementary file 2 [file bmjopen-2025-110204.reviewer_comments.pdf]

PEER REVIEW HISTORY

BMJ Open publishes all reviews undertaken for accepted manuscripts. Reviewers are asked to complete a checklist review form and are provided with free text boxes to elaborate on their assessment. These free text comments are reproduced below.

ARTICLE DETAILS

Title (Provisional)

Protective factors for maternal mental health and life satisfaction during the COVID-19 pandemic: a longitudinal analysis

Authors

Johannessen, Pia Myklebust; Madsen, Christian; Hart, Rannveig Kaldager; Lund, Ingunn Olea; Pettersen, Johanne Hagen; Gustavson, Kristin; Røysamb, Espen; Nesvåg, Ragnar; Brandlistuen, Ragnhild Eek; Ask, Helga

---

VERSION 1 - REVIEW

**Reviewer**	**1**
**Name**	**Khosravi, Mohsen**
**Affiliation**	**Zahedan University of Medical Sciences, Psychiatry and Clinical Psychology**
**Date**	**26-Sep-2025**
**COI**	**None**

I want to begin by expressing my sincere gratitude for the chance to read and critique your manuscript—honestly, it feels like a real privilege. Your research topic is strikingly timely, and it aligns seamlessly with ongoing academic conversations. I hope the observations I've shared below will help sharpen your main points and bolster the paper's overall influence.

ABSTRACT

Your findings are presented clearly, but it might be worth adding a bit more detail on how each protective factor is measured—such as spelling out what exactly "social support" entails or explaining how you evaluated "relationship satisfaction." Even a quick note comparing these results with existing studies would emphasize how your work builds on current knowledge. Plus, a brief nod to the study's limitations and potential research directions could offer a more rounded perspective on both your scope and your contribution to the scientific community.

INTRODUCTION

While you mention that the evidence surrounding certain protective factors is mixed, a short explanation of why it's mixed—or which elements lead to that inconsistency—could give readers a clearer window into the complexities of the field. You touch on "life satisfaction," yet it might help to define it upfront or describe how you measured it, especially for those who aren't familiar with the concept. Including references to more recent studies (up to the time of writing) would further demonstrate that you're fully engaged with the latest scholarship. In addition, highlighting the longitudinal design more directly might underscore how your work uniquely captures shifts over time, especially in the midst of a crisis like the pandemic.

METHODS

You mention excluding participants who didn't have enough data for mean score calculations, but it would be helpful to say more about how you approached missing data in general—did you use any imputation techniques for missing covariates, for example? Also, even though you factor in some covariates (such as maternal age and education level), you might consider reflecting briefly on the role of other confounders, like baseline mental health or socioeconomic status. The reference to deviations listed in Table S1 piqued my curiosity, so offering a short summary of those deviations in the main text could illuminate their broader effect on your study. While it's not standard in every Methods section, a quick mention of possible limitations—like response bias or how findings might not generalize beyond Norway—could also strengthen transparency. Lastly, including the specific R packages or functions used would be a nice touch for reproducibility.

DISCUSSION

You note that exploring causal pathways is important, but going a step further—maybe describing how those protective factors might influence mental health through specific mechanisms—would enrich the conversation around possible future inquiries. It might also be helpful to dive a bit more into why physical activity levels and employment situations vary across different groups, providing a bit of extra context for readers. On top of that, bringing in a discussion of how broader factors (like governmental policies or cultural norms) shape these associations would situate your work in a larger social framework. While you do talk about clinical implications, pointing to concrete interventions that build on your findings might heighten the practical value of your research. Finally, if it's not already covered in the full article, mentioning the statistical significance and effect sizes of your main findings could really help readers grasp how strong those observed associations are.

Thanks again for the opportunity to review your work. I'm impressed by your contribution and look forward to seeing where these insights lead—both in your own continued research and in the wider academic conversation.

**Reviewer**	**2**
**Name**	**Ferraro, Francesco**

Affiliation **University of Derby, Sports and Exercises Science**
Date **11-Oct-2025**
COI **None**

Thank you for submitting your manuscript entitled “Protective factors for maternal mental health and life satisfaction during the COVID-19 pandemic: a longitudinal analysis” to BMJ Open.

This is a well-structured and methodologically rigorous paper addressing a relevant public health topic. The longitudinal design and the use of the Norwegian Mother, Father, and Child Cohort (MoBa) represent strong assets, and the analyses are appropriately conducted. The manuscript is clearly written and presents interesting findings. However, several areas would benefit from clarification, improved focus, and stronger contextualisation to enhance scientific clarity and policy relevance.

Abstract

Please specify the study design explicitly in the opening sentence (e.g., “longitudinal cohort analysis”).

Include one or two key quantitative results (e.g., β, p-value) to strengthen the summary.

The conclusion should be slightly moderated for instance, avoid using “resilient” unless the evidence clearly supports this term.

Introduction

It would be useful to elaborate briefly on why the Norwegian context is important and how this may affect generalisability. This is a key element because the study risks having minimal or no impact worldwide.

The objectives could be sharpened by distinguishing between (1) assessing change in protective factors and (2) evaluating their association with mental health and life satisfaction.

A short paragraph discussing potential mechanisms (e.g., social welfare, family support policies) would improve contextual grounding.

Methods

Clarify the conceptual distinction between Sample 1 and Sample 2, and explain why both were needed.

Provide more detail on how variables were coded and standardised.

The justification for maintaining AUDIT-C despite low reliability should be clearer.

Explain the rationale for the 0.01 significance level, ideally referencing multiple comparison control.

Results

In addition to statistical significance, the authors could discuss practical significance for example, interpret what a $\beta = 0.085$ increase in physical activity represents in real terms. This is a key element that need to be further explore in the whole document as at the moment the clinical impact/application of the research is missing.

Consider providing a concise comparison of which protective factors had the strongest associations with mental distress and life satisfaction.

If any sensitivity analyses showed borderline or divergent results, a short mention in the main text would improve transparency. Also it is necessary to understand what this means in real-life scenarios.

Discussion

The section could be tightened to focus on three main findings:

Stability of protective factors over time; The unique role of employment situation; Implications for public health and maternal support programs.

Consider discussing how Norway's social and economic policies may have mitigated pandemic impacts and thus limit generalizability.

The term "resilience" should be used cautiously, clarify whether this refers to statistical stability or psychological adaptation.

Some paragraphs could be condensed to avoid repetition.

Conclusion

The conclusion is coherent but could be more action-oriented. As for my comments before it feels like the clinical application or if you want the real-life application is missing.

Emphasize the policy implications and how these findings could inform interventions during future crises and how these can inform other countries.

Keep this section concise (3–4 sentences), summarising key take-home points.

Suggest directions for future research, such as causal pathways or cross-country comparative analyses. Help in define what should be do next in line with study`s limitation.

General Notes

The English is fluent and professional; a light stylistic edit could improve flow (e.g., reducing repeated phrases like "protective factors" and "in both groups").

Figures are informative; more descriptive captions (sample size, confidence intervals) would enhance clarity.

Ensure consistent formatting of statistical terms (p, β).

VERSION 1 - AUTHOR RESPONSE

Reviewer 1

Dr. Mohsen Khosravi, Zahedan University of Medical Sciences

I want to begin by expressing my sincere gratitude for the chance to read and critique your manuscript—honestly, it feels like a real privilege. Your research topic is strikingly timely, and it aligns seamlessly with ongoing academic conversations. I hope the observations I've shared below will help sharpen your main points and bolster the paper's overall influence.

Our answer: Thank you, Dr. Mohsen Khosravi, for your insightful feedback and for contributing to the improvement of our manuscript. We appreciate your valuable suggestions, and we have carefully incorporated them to revise our manuscript and hope this enhance the overall impact of our paper.

Abstract

Your findings are presented clearly, but it might be worth adding a bit more detail on how each protective factor is measured—such as spelling out what exactly "social support" entails or explaining how you evaluated "relationship satisfaction." Even a quick note comparing these results with existing studies would emphasize how your work builds on current knowledge.	Thank you for this comment. We have now included a heading "Outcome measures" in the abstract with the following sentences: "*Primary outcomes were mental distress (measured by the eight-item version of the Hopkins Symptom Checklist) and life satisfaction (measured by the Satisfaction With Life Scale). As the first step, we investigated changes in the levels of social support (defined by the number and frequency of social contact), physical activity (average hours of physical activity during a week), employment situation (actively working vs. sick leave or unemployed), alcohol consumption (measured by the Alcohol Use Disorders Identification Test-Consumption) and relationship satisfaction (measured by the five-item version of the Relationship Satisfaction Scale)"* (line numbers 8-14 on page 2).
Plus, a brief nod to the study's limitations and potential research directions could offer a more rounded perspective on both your scope and your contribution to the scientific community.	We have now included the following sentence in the abstract: "*Future studies should investigate additional mitigating factors that may be particularly relevant during global crises and explore the causal relationship between protective factors, mental health and life satisfaction*" (line numbers 33-35 on page 2)

Introduction

While you mention that the evidence surrounding certain protective factors is mixed, a short explanation of why it's mixed—or which elements lead to that inconsistency—could give readers a clearer window into the complexities of the field.	We have now included the following sentence: "*The inconsistent findings might be due to differences in the age of the children, data collection during different pandemic stages and differences in the measurement instruments used" (line numbers 18-20 on page 4).*
You touch on "life satisfaction," yet it might help to define it upfront or describe how you measured it, especially for those who aren't familiar with the concept.	We have now included the following sentences in the introduction: "*Such factors might affect individuals' mental health, often defined as consisting of two related but separate dimensions; well-being and ill-being (Tamnes et al., 2025). Well-being often includes aspects of subjective wellbeing (Keyes, 2002), such as life satisfaction, defined as the cognitive evaluation of one's life as a whole (Diener et al., 1985), while ill-being includes symptoms of mental illness (Keyes, 2005), ranging from mental distress to mental disorders" (line numbers 4-8 on page 4).*
Including references to more recent studies (up to the time of writing) would further demonstrate that you're fully engaged with the latest scholarship	We have now included two studies published in 2025 in the introduction (page 4):<br>Kozai, A. C., Jones, M. A., Borrowman, J. D., Hauspurg, A., Catov, J. M., Kline, C. E., Whitaker, K. M., & Gibbs, B. B. (2025). Patterns of physical activity, sedentary behavior, and sleep across pregnancy before and during two COVID pandemic years. *Midwifery*, *141*, 104268.<br>"*Mothers reported lower levels of physical activity (de Brito et al., 2021; Kozai et al., 2025)"* (line numbers 15-16 on page 4).<br><br>Zhang, X., Xiao, Y., Xu, P., & Dong, S. (2025). Social support and mental health symptoms during the COVID-19 pandemic: A comprehensive meta-analysis unveils limited protective effects. *Journal of Pacific Rim Psychology*, *19*, 18344909251324571.<br>« *[...] social support was associated with fewer symptoms of anxiety and depression* (Ben Salah et al., 2023; Zhang et al., 2025)" (line numbers 23-24 on page 4).
In addition, highlighting the longitudinal design more directly might underscore how your work uniquely captures shifts over time, especially in the midst of a crisis like the pandemic.	We have now highlighted this under the heading "Objectives" with the following sentence: "*Our study contributes to the literature by analyzing longitudinal data from more than 18 000 mothers in the Norwegian Mother, Father, and Child Cohort Study (MoBa), collected before, during, and after the pandemic*" (line numbers 37-39 on page 4).

Methods

You mention excluding participants who didn't have enough data for mean score calculations, but it would be helpful to say more about how you approached missing data in general—did you use any imputation techniques for missing covariates, for example?	Thank you for this comment. There were generally low missing data as presented in Table S1. Specifically for the covariates the percentage of missing varied between 0,1%-1,5%. Consequently, we did not apply any imputation techniques and used complete case analysis. To make this more clear, we have now specified the use of complete case analysis in the existing sentence under the sub-heading "Sensitivity analyses" and clarified where in appendix there is information about how we handled missing data; "*Participants who did not meet our criteria for calculating mean scores (i.e. information on at least 50% of items in a scale) were excluded from the analyses (complete case analysis; see Table S2 in Appendix for information about missing data)*" (line numbers 37-39 on page 7).
Also, even though you factor in some covariates (such as maternal age and education level), you might consider reflecting briefly on the role of other confounders, like baseline mental health or socioeconomic status.	We now see that we did not clearly state our covariates, albeit some of them were mentioned in the statistical analyses section. We have now gathered all information about covariates under the sub-heading "Covariates" and added the following sentence: "*In the regression analyses, we also included a baseline measure (from Q8) of the outcome of interest and a time variable indicating the specific date the mothers responded to Q14 as covariates in the model*" (line numbers 8-10 on page 7).<br>We have also added the role of other confounders not included in the study as a potential limitation under the sub-heading "Methodological considerations": "*Third, there might be other confounding variables affecting the results that we have not measured*"(line numbers 21-22 on page 11).
The reference to deviations listed in Table S1 piqued my curiosity, so offering a short summary of those deviations in the main text could illuminate their broader effect on your study.	We have now included the following sentence: "*See Table S1 in Appendix for a list of deviations, which mostly are specifications of data and variables used and choices regarding the number of analyses conducted" (line numbers 13-14 on page 5).*
While it's not standard in every Methods section, a quick mention of possible limitations—like response bias or how findings might not generalize beyond Norway—could also strengthen transparency.	As we elaborate on these specific limitations in the discussion under the sub-heading "Methodological considerations", due to space limits we have chosen not to comment on limitations in the method section.
Lastly, including the specific R packages or functions used would be a nice touch for reproducibility.	We appreciate this feedback. Details have now been added in the statistical analyses section, and information about where to find details about R packages used to

	conduct sensitivity analyses in the appendix has been more clearly stated in the main text.

Discussion

You note that exploring causal pathways is important, but going a step further—maybe describing how those protective factors might influence mental health through specific mechanisms—would enrich the conversation around possible future inquiries	We appreciate this suggestion. We have now included the following sentence in the conclusion; "*Future research should also explore potential causal pathways linking these factors to mental health and life satisfaction, and investigate the mechanisms through which protective factors exert their influence*" (line numbers 3-5 on page 12).
It might also be helpful to dive a bit more into why physical activity levels and employment situations vary across different groups, providing a bit of extra context for readers.	We have already provided some possible explanations why levels of physical activity and employment situations differ among mothers in the pre-pandemic and pandemic groups.<br>Regarding physical activity: "*This difference may reflect increased flexibility due to remote work or greater engagement in outdoor activities during the pandemic*" (line numbers 10-11 on page 10).<br><br>Regarding employment situation: "*The different findings could be attributed to a different labor market in Norway, or selection bias in the MoBa sample (Biele et al., 2019)*" (line numbers 23-24 on page 10).<br><br>We have now added sentences discussing the clinical importance of the pandemic difference in physical activity: "*However, the actual impact on physical activity levels was modest. The finding indicates that mothers in the pandemic group reported only 0,09 more mean hours of physical activity (a bit more than five minutes) from Q8 to Q14 compared to mothers in the pre-pandemic group*" (line numbers 11-14 on page 10).<br><br>As most of our findings investigating change in protective factors have minor practical implications, we have chosen not to elaborate more about why these factors differ across the groups.
On top of that, bringing in a discussion of how broader factors (like governmental policies or cultural norms) shape these associations would situate your work in a larger social framework.	We have now included the Norwegian context as a potential limitation and discuss how social policies might reduce generalizability to other countries without such governmental support schemes. "*Last, the pandemic situation, restrictions and governmental support schemes in Norway might have differed from other countries, further limiting the generalizability. For instance, individuals who were temporarily laid off during the pandemic received financial support from the Norwegian Labour and Welfare Administration, which might have mitigated some of the*

	negative effects of job loss" (line numbers 28-32 on page 11).<br><br>We have also highlighted in the introduction and conclusion that our study provides knowledge about protective factors specifically in a welfare state like Norway.<br>In the introduction: "*As culture, society and national policies may influence which factors are important to mental distress and life satisfaction, our study adds to the understanding of potential protective factors in Norway, an egalitarian country with a highly developed social welfare system*" (line numbers 39-40 on page 4 and line number 1 on page 5).<br>In the conclusion: "*This study provides valuable insights into protective factors for maternal mental health and life satisfaction within the context of a welfare state*" (line numbers 34-35 on page 11).
While you do talk about clinical implications, pointing to concrete interventions that build on your findings might heighten the practical value of your research.	While we appreciate this suggestion, we have not explored causality in our study. As we do not know the mechanisms behind the findings, we feel it may be too early to specify any concrete interventions now.
Finally, if it's not already covered in the full article, mentioning the statistical significance and effect sizes of your main findings could really help readers grasp how strong those observed associations are.	Thank you for this comment, we have now added the following sentence in the discussion: "*In our study, being actively working emerged as the strongest protective factor compared to other significant factors, with a 51-57% change of a standard deviation. The remaining significant factors demonstrated low to moderate effect sizes, with changes ranging from 3% to 30% of a standard deviation. While these effects may appear small, they can still be clinically meaningful at the population level, particularly because such factors are modifiable through targeted interventions. Consequently, public health policies should aim to enhance social support, physical activity, employment opportunities and relationship satisfaction, as these factors have the potential to improve mental health and life satisfaction at the population level*" (line numbers 40-42 on page 10 and line numbers 1-5 on page 11).

Thanks again for the opportunity to review your work. I'm impressed by your contribution and look forward to seeing where these insights lead—both in your own continued research and in the wider academic conversation.

Reviewer 2

Dr. Francesco Ferraro, University of Derby

Thank you for submitting your manuscript entitled “Protective factors for maternal mental health and life satisfaction during the COVID-19 pandemic: a longitudinal analysis” to BMJ Open.
This is a well-structured and methodologically rigorous paper addressing a relevant public health topic. The longitudinal design and the use of the Norwegian Mother, Father, and Child Cohort (MoBa) represent strong assets, and the analyses are appropriately conducted. The manuscript is clearly written and presents interesting findings. However, several areas would benefit from clarification, improved focus, and stronger contextualisation to enhance scientific clarity and policy relevance.

Our answer: Thank you, Dr. Francesco Ferraro, for taking the time to read and review our paper. We have carefully considered your insightful feedback and have changed and made improvements in the manuscript to address the areas highlighted for clarification, focus, and contextualization. We are grateful for your constructive suggestions, which we believe has contributed significantly to the scientific clarity and policy relevance of the manuscript.

Abstract

Please specify the study design explicitly in the opening sentence (e.g., “longitudinal cohort analysis”).	We have now included a “Design” heading in the abstract and added the following sentence: “*Longitudinal cohort study.*”
Include one or two key quantitative results (e.g., β, p-value) to strengthen the summary.	We have now included two key results in the abstract, including coefficients and 99% confidence intervals.
The conclusion should be slightly moderated for instance, avoid using “resilient” unless the evidence clearly supports this term.	Thank you for the suggestion. We have changed the sentence and omit the word resilient: “*Changes over time in self-reported levels of protective factors were generally consistent among mothers independent of the pandemic. These factors appear to play an equally important role for mental distress and life satisfaction both under ordinary circumstances and during public health crises. Our findings enhance the understanding of how potential protective factors among mothers are associated with mental distress and life satisfaction in the context of a global stressor*” (line numbers 28-32 on page 2).

Introduction

It would be useful to elaborate briefly on why the Norwegian context is important and how this may affect generalisability. This is a key element because the study risks having minimal or no impact worldwide.	Thank you for this comment. We have now included the following sentence under the heading "Objectives": "*As culture, society and national policies may influence which factors are important to mental distress and life satisfaction, our study adds to the understanding of potential protective factors in Norway, an egalitarian country with a highly developed social welfare system*" (line numbers 39-40 on page 4 and line number 1 on page 5).<br><br>How the Norwegian context may affect generalizability are further elaborated under "Methodological considerations": "*Last, the pandemic situation, restrictions and governmental support schemes in Norway might have differed from other countries, further limiting the generalizability. For instance, individuals who were temporarily laid off during the pandemic received financial support from the Norwegian Labour and Welfare Administration, which might have mitigated some of the negative effects of job loss*" (line numbers 28-32 on page 11).
The objectives could be sharpened by distinguishing between (1) assessing change in protective factors and (2) evaluating their association with mental health and life satisfaction.	We have now tried to sharpen the aims to distinguish the two overall objectives (line numbers 3-9 on page 5):<br>1. ***Assessing change in protective factors**: How did the levels of five putative protective factors—social support, physical activity, employment situation, alcohol consumption, and relationship satisfaction—change during the pandemic compared to pre-pandemic times?*<br>2. ***Evaluating associations between protective factors and mental health and life satisfaction:** How are these protective factors associated with mental distress and life satisfaction, and do these associations differ between mothers exposed to the pandemic and those who were not?*
A short paragraph discussing potential mechanisms (e.g., social welfare, family support policies) would improve contextual grounding.	We have addressed this issue in your comment above regarding the relevance of the Norwegian context. "*As culture, society and national policies may influence which factors are important to mental distress and life satisfaction, our study adds to the understanding of potential protective factors in Norway, an egalitarian country with a highly developed social welfare system*" (line numbers 39-40 on page 4 and line number 1 on page 5).

Methods

Clarify the conceptual distinction between Sample 1 and Sample 2, and explain why both were needed.	Thank you for this comment. In the revised manuscript we have explained the distinction between Sample 1 and 2, and why both were needed: "*Two analytical samples were defined based on the statistical analyses used to examine the two research questions. Both samples were restricted to mothers responding to Q8 and Q14 (n=18 339, Sample 2). In order to examine within-person pandemic effects on protective factors, Sample 1 (n=18 015) was further restricted to make the data waves more comparable. Due to a delay in the initiation of the Q14 data collection, Q14 data was collected over a 7-year period (2017-2023). Consequently, we also restricted Q8 data to responses collected between 2011–2017 (7 years). Q14 mothers who (due to the delay) responded when their child was older than 15 years old in Q14 were excluded from Sample 1" (line numbers 33-40 on page 5).*
Provide more detail on how variables were coded and standardised.	Thank you for this feedback. Detailed information about variables and how they are coded is described in detail in the appendix. However, we have now provided a specific reference to the appendix in the main text to make this information more available: "*For more details about the preparation of the mental health outcome variables see Appendix section 2*" (line number 9 on page 6). "*For more details about the preparation of the protective factors see Appendix section 2*" (line number 32 on page 6).<br>We have also provided information about how variables are standardized in the methods section under the sub-heading "Statistical analyses", as this previously was only described in the appendix: "*All outcomes and predictors were standardized to have a mean of 0 and standard deviation of 1" (line numbers 28-29 on page 7)*
The justification for maintaining AUDIT-C despite low reliability should be clearer.	We decided to use AUDIT-C as a continuous variable to measure mothers' alcohol consumption, despite low reliability. To justify this choice, we have now added the following sentence under the heading sensitivity analyses: "*AUDIT-C showed a low reliability, which might indicate that the items included in AUDIT-C measure different aspects of alcohol consumption. Despite low reliability, we still believe that the total score of these different aspects provides a useful measure of alcohol consumption. However, to test whether the low reliability led to an underestimation of the associations, we performed separate regression analyses including only the first item as predictor"* (line number 42 on page 7 and line numbers 1-5 on page 8).
Explain the rationale for the 0.01 significance level, ideally referencing multiple comparison control.	The rationale is explained in the section of statistical analyses: "*The significance level in all analyses were set to 0.01, with corresponding 99% confidence intervals (99% CI). A conservative p-value threshold was chosen, to reduce the*

	risk of type I errors due to multiple comparisons" (line numbers 31-33 on page 7)

Results

In addition to statistical significance, the authors could discuss practical significance for example, interpret what a β = 0.085 increase in physical activity represents in real terms.	We have added the following sentence in the Discussion section: "*However, the actual impact on physical activity levels was modest. The finding indicates that mothers in the pandemic group reported only 0,09 more mean hours of physical activity (a bit more than five minutes) from Q8 to Q14 compared to mothers in the pre-pandemic group*" (line numbers 11-14 on page 10).
This is a key element that need to be further explore in the whole document as at the moment the clinical impact/application of the research is missing.	Thank you for this comment. We have now included the following paragraph in the discussion: "*In our study, being actively working emerged as the strongest protective factor compared to other significant factors, with a 51-57% change of a standard deviation. The remaining significant factors demonstrated low to moderate effect sizes, with changes ranging from 3% to 30% of a standard deviation. While these effects may appear small, they can still be clinically meaningful at the population level, particularly because such factors are modifiable through targeted interventions. Consequently, public health policies should aim to enhance social support, physical activity, employment opportunities and relationship satisfaction, as these factors have the potential to improve mental health and life satisfaction at the population level*" (line numbers 40-42 on page 10 and line numbers 1-5 on page 11).
Consider providing a concise comparison of which protective factors had the strongest associations with mental distress and life satisfaction.	We appreciate this suggestion. However, the aim of the paper was to examine which of the five selected protective factors were associated with mental distress and life satisfaction. Statistically comparing which protective factors had the strongest associations with mental distress and life satisfaction was not a part of our preregistration: https://osf.io/4zvpj/?view_only=. We believe it is sufficient to compare standardized estimates, as this gives an indication of which predictors showed a stronger association with mental distress and life satisfaction.
If any sensitivity analyses showed borderline or divergent results, a short mention in the main text would improve transparency. Also it is necessary to understand what this means in real-life scenarios.	Thank you for this comment, we have now elaborated on the borderline significant alcohol sensitivity analysis for mental distress, and what this means in real-life scenarios: "*The different results for mental distress and life satisfaction show the complexity when measuring alcohol consumption in this sample and indicate that mental distress and life satisfaction are related to the different aspects of alcohol consumption measured in AUDIT-C to different degrees*" (line numbers 29-33 on page 9).

Discussion

The section could be tightened to focus on three main findings: Stability of protective factors over time; The unique role of employment situation; Implications for public health and maternal support programs.	We consider the main findings to be 1) the lack of pandemic differences in protective factors over time; 2) the similar protective factors for both mental distress and life satisfaction; and 3) the finding of only one moderating effect of pandemic exposure, which was on the association between employment situation and mental distress. Consequently, the discussion section is organized to focus on these three findings.
Consider discussing how Norway's social and economic policies may have mitigated pandemic impacts and thus limit generalizability.	Thank you for this comment. We have now added the following sentences under methodological considerations: "*Last, the pandemic situation, restrictions and governmental support schemes in Norway might have differed from other countries, further limiting the generalizability. For instance, individuals who were temporarily laid off during the pandemic received financial support from the Norwegian Labour and Welfare Administration, which might have mitigated some of the negative effects of job loss"* (line numbers 28-32 on page 11).
The term "resilience" should be used cautiously, clarify whether this refers to statistical stability or psychological adaptation.	We appreciate this comment. We have now rephrased the two existing sentences using resilience in the discussion and conclusion sections.
Some paragraphs could be condensed to avoid repetition.	Thank you for this comment. Due to the suggestions for improvements from both reviewers, the discussion has been condensed. We hope that the new text is not experienced as repetitive.

Conclusion

The conclusion is coherent but could be more action-oriented. As for my comments before it feels like the clinical application or if you want the real-life application is missing.	Thank you for this comment. We have now rephrased the conclusion section and tried to make it more action oriented. "*This study provides valuable insights into protective factors for maternal mental health and life satisfaction within the context of a welfare state. The similar levels of social support, employment situation, alcohol consumption and relationship satisfaction among all mothers over time, suggest that these factors are more robust to external stressors like a global pandemic. Importantly, these factors appear to play a consistently significant role in relation to mental distress and life satisfaction, both under ordinary circumstances and during public health crises. However, the heightened significance of employment status during the pandemic*

	highlights its potential role as a buffer in times of economic uncertainty. Our findings can guide the development of targeted interventions to support maternal mental health and life satisfaction, by focusing on enhancing social support, physical activity, employment opportunities and relationship satisfaction. Future large-scale studies are needed to identify additional protective factors that may be particularly relevant during global crises. Future research should also explore potential causal pathways linking these factors to mental health and life satisfaction, and investigate the mechanisms through which protective factors exert their influence" (line numbers 34-42 on page 11 and line numbers 1-5 on page 12).
Emphasize the policy implications and how these findings could inform interventions during future crises and how these can inform other countries.	We have now specifically pointed to factors that should be targeted in the development of interventions; "*Our findings can guide the development of targeted interventions to support maternal mental health and life satisfaction, by focusing on enhancing social support, physical activity, employment opportunities and relationship satisfaction*" (line numbers 41-42 on page 11 and line number 1 on page 12).
Keep this section concise (3–4 sentences), summarising key take-home points.	We appreciate this feedback. However, due to suggestions for being more action-oriented and including more information about policy implications and directions for future research (from both reviewers), we found it difficult to stay within 3-4 sentences and ended up with 7 sentences.
Suggest directions for future research, such as causal pathways or cross-country comparative analyses. Help in define what should be do next in line with study`s limitation.	We have now rephrased the directions for future research: "*Future large-scale studies are needed to identify additional protective factors that may be particularly relevant during global crises. Future research should also explore potential causal pathways linking these factors to mental health and life satisfaction, and investigate the mechanisms through which protective factors exert their influence*" (line numbers 1-5 on page 12).

General Notes

The English is fluent and professional; a light stylistic edit could improve flow (e.g., reducing repeated phrases like "protective factors" and "in both groups").	We have now reduced repeated phrases.
Figures are informative; more descriptive captions (sample size, confidence	Thank you for this comment. We have now included 99% Confidence intervals in the figure captions. We appreciate the suggestion of including sample size in figure captions, as we agree that this would enhance clarity for the reader. However,

intervals) would enhance clarity.	most figures present results from several separate analyses, each with its distinct sample size. Including all different sample sizes directly in the figure caption could reduce the clarity and readability of the information provided. As sample sizes are included in the tables in Appendix, we consider it best not to include this information in the figure caption.
Ensure consistent formatting of statistical terms (p, β).	We appreciate this comment. While we understand that this seems inconsistent, the reason for using both B and β, is that the results regarding change in protective factors are not standardized, while the results regarding the associations between protective factors and mental health outcomes are standardized.

VERSION 2 - REVIEW

Reviewer 1

Name Khosravi, Mohsen

Affiliation Zahedan University of Medical Sciences, Psychiatry and Clinical Psychology

Date 13-Nov-2025

COI

Thank you very much for taking the time to carefully consider and address all of my comments and suggestions. I truly appreciate your thorough attention to detail and responsiveness, as it greatly contributes to improving the overall quality of our work and ensures effective communication and collaboration between us.

Reviewer 2

Name Ferraro, Francesco

Affiliation University of Derby, Sports and Exercises Science

Date 08-Dec-2025

COI

This manuscript examines whether five established protective factors, social support, physical activity, employment situation, alcohol consumption, and relationship satisfaction, changed during the COVID-19 pandemic and how these factors related to maternal mental distress and life satisfaction. The study draws on data from approximately 18,000 mothers participating in the Norwegian Mother, Father and Child Cohort Study (MoBa), who completed pre-pandemic (Q8) and mid-pandemic (Q14) questionnaires.

This is a well-written manuscript that leverages a rich longitudinal dataset and applies appropriate analytic methods. The preregistered plan, transparent reporting, and thoughtful discussion enhance the study's credibility. Some conceptual and methodological clarifications would further strengthen the manuscript.

1. Conceptual framing

The introduction effectively summarises existing research but could more clearly articulate the conceptual rationale for treating these variables as "protective factors." While prior associations with mental health are described, anchoring the selection in a theoretical framework (e.g., stress-buffering, family resilience, behavioural regulation models) would sharpen the scientific contribution. Additionally, the study focuses on mothers of children aged 8 and 14. This distinguishes it from research on perinatal maternal mental health, and providing more explicit justification for why these developmental stages matter would increase conceptual clarity.

2. Definition of pandemic exposure and timing considerations

The manuscript accurately states that Q14 data were collected between 2017 and 2023 and that pandemic exposure is determined solely by whether responses occurred before or after the March 2020 lockdown. While this classification is valid, it may introduce potential confounding by trends unrelated to the pandemic. Further discussion of this issue, particularly regarding assumptions, would be beneficial.

3. Measurement of protective factors

Several measurement decisions should be described more fully:

• Social support: The two items capture different dimensions (availability and frequency). Clarifying their content more explicitly in the Methods would help readers interpret the composite measure.

• Physical activity: Combining three weekly duration categories into a continuous score is sensible, but a brief justification for treating them as additive and linear would increase transparency.

• Employment status: The DID analyses treat unemployment and sick leave as distinct contrasts; however, regression models combine them into a single "not working" category. Given that unemployment and sickness absence may reflect different underlying mechanisms, further justification for this analytic choice is warranted.

4. Analytical approach

The DID and RDD approaches are appropriate, but several vital details require clarification:

• Parallel trends assumption: The DID design relies on this assumption, yet no graphical or descriptive support is provided. Even a brief conceptual justification for assuming parallel pre-pandemic trends would strengthen the approach's validity.

• Regression discontinuity limitations: The RDD for relationship satisfaction (due to lack of a Q8 measure) estimates only the effect at the lockdown cutoff. Stating this explicitly in the Methods would help set expectations for interpreting the null finding.

• Missing data: Complete-case analysis is noted, and missingness rates appear in the appendix, but adding a brief justification for this approach in the main text would improve transparency.

5. Interpretation of results

The Results section is clear and well presented. A few points merit deeper interpretation:

• Physical activity DID effect: While statistically significant, the impact (~0.085 hours/week, ≈ 5 minutes) is minimal. Explicitly discussing its practical significance—or lack thereof—would help contextualise its importance.

• Baseline differences in social support: Pandemic-exposed and pre-pandemic groups differ slightly at Q8 (minimal effect). A comment on how this relates to DID assumptions would be helpful.

• Employment moderation: The stronger association between employment and mental distress during the pandemic is a central finding. Quantifying this effect relative to the main employment effect discussed in the Discussion would make its implications clearer.

6. Discussion

The Discussion thoughtfully integrates findings with existing literature. A few refinements would strengthen it:

• Avoiding causal interpretations: Some recommendations read as causal (e.g., policies "should enhance social support"), though the regression models are cross-sectional. Slightly more cautious language would reflect the analytic limitations.

• Role of Norwegian welfare supports: The manuscript notes that the Norwegian welfare system may buffer employment-related stress. Expanding this point would enhance readers' understanding of the context in which these results should be interpreted.

• Multicollinearity: Some protective factors (e.g., social support and relationship satisfaction) may be correlated. Including a brief note on whether multicollinearity diagnostics were examined would bolster confidence in the regression estimates.

Minor Comments

• The Abstract could clarify that pandemic grouping was based on questionnaire response timing, not cohort recruitment.

• Figure 1 uses different metrics across panels (linear coefficients vs odds ratios). A clarifying note in the figure legend would help readers interpret the results correctly.

• Figure 2 is informative but could benefit from including numeric beta values alongside graphical estimates.

• A few minor typographical issues appear in the Results section (e.g., missing letters or spacing).

• Some early Discussion paragraphs repeat results already stated; slight condensation may improve flow.

VERSION 2 - AUTHOR RESPONSE

Reviewer: 1

Dr. Mohsen Khosravi, Zahedan University of Medical Sciences
Comments to the Author:
Thank you very much for taking the time to carefully consider and address all of my comments and suggestions. I truly appreciate your thorough attention to detail and responsiveness, as it greatly contributes to improving the overall quality of our work and ensures effective communication and collaboration between us.

Our answer: Thank you, Dr. Mohsen Khosravi, for the positive comments.

Reviewer: 2

Dr. Francesco Ferraro, University of Derby
Comments to the Author:
This manuscript examines whether five established protective factors, social support, physical activity, employment situation, alcohol consumption, and relationship satisfaction, changed during the COVID-19 pandemic and how these factors related to maternal mental distress and life satisfaction. The study draws on data from approximately 18,000 mothers participating in the Norwegian Mother, Father and Child Cohort Study (MoBa), who completed pre-pandemic (Q8) and mid-pandemic (Q14) questionnaires.
This is a well-written manuscript that leverages a rich longitudinal dataset and applies appropriate analytic methods. The preregistered plan, transparent reporting, and thoughtful discussion enhance the study's credibility. Some conceptual and methodological clarifications would further strengthen the manuscript.

Our answer: Thank you, Dr. Francesco Ferraro, for your insightful feedback. Based on the second round of revisions, we have now changed and made improvements in the manuscript to address conceptual and methodological considerations. We are grateful for your constructive suggestions, which we believe has contributed to strengthening the manuscript.

1. Conceptual framing

The introduction effectively summarises existing research but could more clearly articulate the conceptual rationale for treating these variables as "protective factors."<br><br>While prior associations with mental health are described, anchoring the selection in a theoretical framework (e.g., stress-buffering, family resilience, behavioural regulation models) would sharpen the scientific contribution.	Thank you for these comments. We have now rewritten the second paragraph of the introduction to both articulate the conceptional rationale for treating these as protective factors and anchored the selected factors in existing theoretical frameworks to increase the scientific contribution. New text (see page 4, lines 13-29): *"The emergence of resilience research and later positive psychology in the mid-1990s marked a shift in the scientific literature from symptom-focused medical models to positive outcomes and protective factors [8]. Drawing on Bronfenbrenner's ecological systems theory [9], protective factors can be understood as resources within various levels of an individual's ecological environment that reduce negative effects or enhance positive outcomes. Existing literature highlights several protective factors for mental health, including social support, physical activity, employment, lower alcohol consumption, and relationship satisfaction [10-12]. These protective factors are also anchored in other established theoretical frameworks. For example, the Transactional Theory of Stress and Coping [13, 14] highlight the role of emotion- and problem-focused coping, which could include using social support, physical activity and reducing alcohol intake to regulate emotions and handle stress, while the family resilience framework [15, 16] emphasizes relational processes such as relationship satisfaction to help families adapt to adversity. However, the pandemic may have disrupted such protective factors. For example, mothers reported reduced physical activity [17, 18], and increased experiences of job loss or anticipated job loss [19] during the pandemic. Evidence regarding social support [20, 21], alcohol use [22], and relationship satisfaction [23, 24] among mothers is mixed, potentially due to variations in child age, timing of data collection across different pandemic phases, and differences in the measurement instruments used."*
Additionally, the study focuses on mothers of children aged 8 and 14. This distinguishes it from research on perinatal maternal mental health, and providing more explicit justification for why these developmental stages matter would increase conceptual clarity.	Thank you for this comment. We have now revised the last paragraph in the introduction (lines 41-42 on page 4 and lines 1-8 on page 5). New text now reads: *"Importantly, much of the existing research has focused on perinatal or postpartum mothers, leaving a gap in understanding mothers of older children. The developmental stages of childhood and adolescence introduce distinct challenges compared to early parenthood. As children mature, increasing autonomy, academic demands, and social complexity can heighten parental stress and require different coping strategies. Mothers at these stages often balance caregiving with employment and household responsibilities, making protective factors such as social support, relationship satisfaction, and behavioral regulation particularly relevant. Additionally, few studies have used life satisfaction as a key outcome alongside mental distress when investigating pandemic effects among mothers, and many lack pre-pandemic baseline measures, limiting the ability to assess changes over time."*

2. Definition of pandemic exposure and timing considerations

The manuscript accurately states that Q14 data were collected between 2017 and 2023 and that pandemic exposure is determined solely by whether responses occurred before or after the March 2020 lockdown. While this classification is valid, it may introduce potential confounding by trends unrelated to the pandemic. Further discussion of this issue, particularly regarding assumptions, would be beneficial.	We have now included this as a potential methodological limitation in the discussion (page 12, lines 8-13): *"Fifth, since Q14 data was collected over an extended period (2017-2023), classification into pre-pandemic and pandemic groups may partly capture secular trends unrelated to the COVID-19 pandemic. Our analyses therefore rely on the assumption that underlying time trends in protective factors and mental health outcomes would have evolved similarly in the absence of the pandemic. However, residual confounding by unmeasured time-varying factors cannot be excluded."*

3. Measurement of protective factors
Several measurement decisions should be described more fully:

Social support: The two items capture different dimensions (availability and frequency). Clarifying their content more explicitly in the Methods would help readers interpret the composite measure.	We have revised the text concerning assessment of social support and included detailed descriptions of the two items in the methods section (page 6, lines 26-28):<br><br>*"Two items assessed social support, measuring both availability (having someone other than a partner to seek advice from in a difficult situation) and* frequency (how often mothers meet or talk with their extended family or close friends). *Each item was scored on a three-point scale, ranging from 1 to 3."*<br><br>A detailed description of the included items is also available in the appendix (section 2.2).
Physical activity: Combining three weekly duration categories into a continuous score is sensible, but a brief justification for treating them as additive and linear would increase transparency.	We have included a justification for treating the physical activity items as additive and linear in the Appendix (section 2.2): "*We operationalized physical activity as a continuous variable by summing three non-overlapping weekly duration categories and dividing by number of answered items, to calculate the mean hours of moderate-to-vigorous physical activity per week. This approach assumes an approximately linear and monotonic relationship between weekly exercise duration and mental health outcomes, an assumption widely adopted in epidemiological research. Treating physical activity as continuous facilitates interpretability and comparability across studies."*

Employment status: The DID analyses treat unemployment and sick leave as distinct contrasts; however, regression models combine them into a single “not working” category. Given that unemployment and sickness absence may reflect different underlying mechanisms, further justification for this analytic choice is warranted.	In the regression analyses, we examined associations between protective factors and either mental distress or life satisfaction. Although unemployment and sick leave may reflect different underlying mechanisms, both represent absence from paid work and have consistently been associated with poorer mental health outcomes [1-4]. Given these findings, we decided to combine these two categories into a single category labelled 'not working', as both being on sick leave and unemployment implies that mothers are not currently working and entail worse mental health. This choice adheres to the aim of our paper, as we were interested in examining the protective effect of being actively working on levels of mental distress and life satisfaction compared to not being actively working.<br><br>We have now revised the text in the methods section (page 6, line 39-42): *“When examining the potential protective effect of being actively working on levels of mental distress and life satisfaction in the regression models, we created a binary variable separating those currently employed vs. those on sick leave/unemployed.”*<br><br>[1] Amiri, S., & Behnezhad, S. (2021). Depression symptoms and risk of sick leave: a systematic review and meta-analysis. *International archives of occupational and environmental health*, *94*(7), 1495-1512.<br>[2] Gjesdal, S., Ringdal, P. R., Haug, K., & Mæland, J. G. (2004). Predictors of disability pension in long-term sickness absence: results from a population-based and prospective study in Norway 1994–1999. *The European Journal of Public Health*, *14*(4), 398-405.<br>[3]Virgolino, A., Costa, J., Santos, O., Pereira, M. E., Antunes, R., Ambrosio, S., ... & Vaz Carneiro, A. (2022). Lost in transition: a systematic review of the association between unemployment and mental health. *Journal of Mental Health*, *31*(3), 432-444.<br>[4]Reneflot, A., & Evensen, M. (2014). Unemployment and psychological distress among young adults in the n ORDIC countries: A review of the literature. *International Journal of Social Welfare*, *23*(1), 3-15.

4. Analytical approach

The DID and RDD approaches are appropriate, but several vital details require clarification:

Parallel trends assumption: The DID design relies on this assumption, yet no graphical or descriptive support is provided. Even a brief conceptual justification for assuming parallel pre-pandemic trends would strengthen the approach's validity.	The parallel trends assumption states that in the absence of an intervention or exposure, the two groups (intervention and control groups) would have a parallel/constant development over time. As this assumption implies that the intervention group (in our case the mothers who responded during the pandemic) never receives an intervention, this assumption can never be formally tested, because the intervention group receives an intervention or exposure at follow up. Some studies test this assumption indirectly, by examining trends between the two groups over time before an intervention or exposure. If the trends are parallel in the time before the intervention or exposure, one assumes that this trend would have continued in the absence of an intervention or exposure. However, this requires several measurements of both groups before the intervention or exposure occurs.

	Since our study used a simple DID analysis with only two time points, it is not possible for us to examine trends before Q8. Instead, one assumes that group differences found in Q8 remain constant over time (Q14) and that factors that can vary with time, from Q8 to Q14, have a similar effect on the two groups. It is these two assumptions that together form the parallel trends assumption, as this will lead to a constant development over time in the absence of an intervention or exposure. Although we cannot empirically test parallel pre-trends due to having only two time points, the assumption is plausible in our setting because group assignment is determined solely by response timing rather than individual characteristics. The pandemic and associated lockdown represent an external, population-wide stressor, and the two groups were comparable at baseline (Q8) with respect to observed characteristics. Moreover, sensitivity analyses using Q8 as a negative control in the regression analyses did not indicate differential trends to the pandemic, lending indirect support to the parallel trends assumption.<br><br>We have now included the following sentences in the limitations (page 12, lines 5-8): "*Fourth, the DID analyses rely on the parallel trends assumption, which cannot be empirically tested in our design due to having only two time points. However, because group assignment is determined solely by response timing and the pandemic represents an external, population-wide stressor, we consider the assumption plausible*."
Regression discontinuity limitations: The RDD for relationship satisfaction (due to lack of a Q8 measure) estimates only the effect at the lockdown cutoff. Stating this explicitly in the Methods would help set expectations for interpreting the null finding.	Thank you for this suggestion. We have now revised the text in the Method section accordingly (page7, line 33-35) : "*Due to the lack of a Q8 measure for relationship satisfaction, we used regression discontinuity design (RDD) to investigate an immediate change in level of relationship satisfaction between the pre-pandemic and pandemic groups at the Norwegian lockdown (see details in Appendix section 5.2).*"
Missing data: Complete-case analysis is noted, and missingness rates appear in the appendix, but adding a brief justification for this approach in the main text would improve transparency.	We have now added the following sentence in the statistical analyses section (page 8, lines 8-11): "*Due to a large sample size and no major differences between the analytic sample and the full MoBa sample (see Table S3 in Appendix), we used complete-case analysis. This approach assumes that missingness is not strongly related to the outcomes conditional on observed covariates, and sensitivity analyses yielded similar results*."

5. Interpretation of results
The Results section is clear and well presented. A few points merit deeper interpretation:

Physical activity DID effect: While statistically significant, the impact (~0.085 hours/week, ≈ 5 minutes) is minimal. Explicitly discussing its practical significance—or lack thereof—would help contextualise its importance.	We have now revised the existing sentences in the discussion (page 10, lines 29-33): "*While statistically significant, the actual impact on physical activity levels was minimal. The finding indicates that mothers in the pandemic group reported only 0,09 more mean hours of physical activity (a bit more than five minutes) from Q8 to Q14 compared to mothers in the pre-pandemic group. This small difference is unlikely to be of practical relevance at the individual level*."
Baseline differences in social support: Pandemic-exposed and pre-pandemic groups differ slightly at Q8 (minimal effect). A comment on how this relates to DID assumptions would be helpful.	The parallel trends assumption does not require the two groups to have identical baseline levels, only that any baseline differences remain stable over time (from Q8 to Q14). Although the pandemic-exposed and pre-pandemic groups differed slightly in social support at Q8, the magnitude of this difference was small and is therefore unlikely to bias the estimated DID effect.
Employment moderation: The stronger association between employment and mental distress during the pandemic is a central finding. Quantifying this effect relative to the main employment effect discussed in the Discussion would make its implications clearer.	Table S12 show that the interaction effect had a relatively low effect size, with approximately 10% change of a standard deviation, relative to the 51% change of a standard deviation for the main finding. We have now tried to quantify this effect, by calculating the difference in SCL-8 scores shown in Figure 3. Based on the scores, we can see that the protective effect of working corresponds to 0,31 lower symptoms in SCL-8 among mothers responding before the pandemic and 0,44 lower symptoms among mothers responding during the pandemic.<br><br>We have elaborated on what this finding means in the discussion section (page 11, lines 25-36): "*When investigating whether pandemic exposure moderated these associations, we found that the association between employment situation and mental distress was stronger within the pandemic group compared to the pre-pandemic group. As shown in Figure 3, the protective effect of working corresponds to 0,31 lower SCL-8 score among mothers responding before the pandemic and 0,44 among mothers responding during the pandemic. This difference could be related to the uncertainty regarding the employment situation, especially during the early stages of the pandemic. Being temporarily or permanently laid off has serious consequences for household income, which could explain why being actively working was more important for the mental health of mothers during the pandemic compared to the pre-pandemic group of mothers. Being actively working during the pandemic could also indicate that the mothers were less isolated. While the clinical impact of this finding may be minimal on an individual level, even small reductions in symptoms*

	of mental distress across a population could be important for public health efforts."

6. Discussion
The Discussion thoughtfully integrates findings with existing literature. A few refinements would strengthen it:

Avoiding causal interpretations: Some recommendations read as causal (e.g., policies "should enhance social support"), though the regression models are cross-sectional. Slightly more cautious language would reflect the analytic limitations.	We appreciate this feedback. We have now revised two existing sentences in the discussion and conclusion to avoid language implying causation:<br>"*Consequently, public health policies may aim to enhance social support, physical activity, employment opportunities and relationship satisfaction, as these factors have the potential to improve mental health and life satisfaction at the population level*." (page 11, lines 22-24)<br><br>"*Our findings may guide the development of targeted interventions to support maternal mental health and life satisfaction, by focusing on enhancing social support, physical activity, employment opportunities and relationship satisfaction*" (page 12, lines 34-36)
Role of Norwegian welfare supports: The manuscript notes that the Norwegian welfare system may buffer employment-related stress. Expanding this point would enhance readers' understanding of the context in which these results should be interpreted.	Receiving financial support if one were temporarily laid off during the pandemic may buffer employment-related stress, and such information would enhance readers understanding of the Norwegian context when interpretating the results. We have now elaborated this point and added a sentence in the limitations (page 12, lines 23-25): "*This context should be considered when interpreting the strength of the observed associations and their generalizability to settings with less comprehensive welfare systems*."
Multicollinearity: Some protective factors (e.g., social support and relationship satisfaction) may be correlated. Including a brief note on whether multicollinearity diagnostics were examined would bolster confidence in the regression estimates.	Thank you for this comment. We have now included the following sentence in Appendix section 7: "*We assessed multicollinearity among protective factors prior to the regression analyses. Variance inflation factors (VIFs) ranged from 1 to 3 (with response date and pandemic group at the upper end), and pairwise correlations were low (r = 0.01-0.09), well below commonly used thresholds, indicating no problematic multicollinearity*."

Minor Comments

The Abstract could clarify that pandemic grouping was based on questionnaire response timing, not cohort recruitment.	We have written in the abstract that pandemic grouping was based on responses and not cohort recruitment.<br>"*We analyzed data from two waves of the Norwegian Mother, Father, and Child Cohort Study (n = ~18 000 mothers); one pre-pandemic wave and one wave in which half of the sample responded after the onset of the pandemic, with pandemic exposure defined by questionnaire response timing rather than cohort recruitment." (page 2, lines 15-18)*
Figure 1 uses different metrics across panels (linear coefficients vs odds ratios). A clarifying note in the figure legend would help readers interpret the results correctly.	Thank you for this comment. This clarification was previously only stated in the y-axis, but we have now included a sentence in the Figure legend: "*Panels A, B, E and F show regression coefficients, and panels C and D show odds ratios*".
Figure 2 is informative but could benefit from including numeric beta values alongside graphical estimates.	The specific values of standardized coefficients are found in Tables S12-S13 in the Appendix. We have included a sentence in the Figure legend clarifying that the x-axis shows standardized coefficients: "*The x-axis shows standardized coefficients. Exact standardized coefficients and confidence intervals are provided in Tables S12-S13 in the Appendix*".
A few minor typographical issues appear in the Results section (e.g., missing letters or spacing).	Thank you for noting this. We have carefully reviewed the Results section and corrected the identified typographical errors.
Some early Discussion paragraphs repeat results already stated; slight condensation may improve flow.	We believe you are referring to the summary of main findings in the first paragraph of the discussion. We prefer to keep this section.

VERSION 3 - REVIEW

**Reviewer**	**2**
**Name**	**Ferraro, Francesco**
**Affiliation**	**University of Derby, Sports and Exercises Science**
**Date**	**06-Jan-2026**
**COI**	

Thank you for responding to my comments and feedback. To the best of my knowledge, this paper makes an interesting contribution to the field; therefore, I am happy to see it accepted.
